# Stage-Specific Genetic Interaction between *FgYCK1* and *FgBNI4* during Vegetative Growth and Conidiation in *Fusarium graminearum*

**DOI:** 10.3390/ijms23169106

**Published:** 2022-08-14

**Authors:** Jindong Zhu, Denghui Hu, Qianqian Liu, Rui Hou, Jin-Rong Xu, Guanghui Wang

**Affiliations:** 1 State Key Laboratory of Crop Stress Biology for Arid Areas, College of Plant Protection, Northwest A&F University, Yangling, Xianyang 712100, China; 2 College of Forestry, Guizhou University, Guiyang 550025, China; 3 Department of Botany and Plant Pathology, Purdue University, West Lafayette, IN 47907, USA

**Keywords:** *Gibberella zeae*, polarized growth, cell wall integrity, wheat scab fungus, Fusarium head blight

## Abstract

CK1 casein kinases are well conserved in filamentous fungi. However, their functions are not well characterized in plant pathogens. In *Fusarium graminearum*, deletion of *FgYCK1* caused severe growth defects and loss of conidiation, fertility, and pathogenicity. Interestingly, the *Fgyck1* mutant was not stable and often produced fast-growing spontaneous suppressors. Suppressor mutations were frequently identified in the *FgBNI4* gene by sequencing analyses. Deletion of the entire *FgBNI4* or disruptions of its conserved C-terminal region could suppress the defects of *Fgyck1* in hyphal growth and conidiation, indicating the genetic relationship between *FgYCK1* and *FgBNI4*. Furthermore, the *Fgyck1* mutant showed defects in polarized growth, cell wall integrity, internalization of FgRho1 and vacuole fusion, which were all partially suppressed by deletion of *FgBNI4*. Overall, our results indicate a stage-specific functional relationship between *FgYCK1* and *FgBNI4*, possibly via FgRho1 signaling for regulating polarized hyphal growth and cell wall integrity.

## 1. Introduction

The homothallic ascomycete *Fusarium graminearum* is one major causal agent of the destructive Fusarium head blight (FHB) disease on wheat and barley [1,2]. Ascospores released from perithecia that overwinter on infected plant debris are the primary inoculum of FHB [3,4]. This important fungal pathogen not only causes severe yield loss but also produces harmful mycotoxins in infected kernels, including deoxynivalenol (DON) and zearalenone (ZEA) [5,6]. As a potent protein biosynthesis inhibitor, DON is also an important virulence factor during plant infection.

In a previous study, we systematically characterized the 116 protein kinases in *F. graminearum* [7]. Among the 96 protein kinase genes with knockout mutants identified and characterized in *F. graminearum*, several are single copy genes that are orthologous to two paralogs in the budding yeast *Saccharomyces cerevisiae*. In yeast, deletion of these paralogous kinase genes individually, such as *YCK1* and *YCK2* or *TOR1* and *TOR2*, is not lethal but the double mutants are inviable. In *F. graminearum*, deletion of the only TOR kinase appears to be lethal but deletion of the only ortholog of *YCK1* and *YCK2* is viable; however, the *Fgyck1* deletion mutant generated in the systematic characterization of the *F. graminearum* kinome has severe growth defects [7]. Moreover, FgYck1 also interacts with the HOPS tethering complex subunit FgVps41 and regulates vacuole membrane fusion [8].

Casein kinases are highly conserved serine/threonine protein kinases that were firstly characterized in rat livers and named for their preferential utilization of casein and other acidic proteins [9]. Casein kinases can be divided into two subtypes: casein kinase I (CK1) and casein kinase II (CK2). CK2 isoforms are usually found as a heterotetramer, whereas CK1 isoforms are functional as a monomeric enzyme [10,11]. CK1 is ubiquitously expressed in all eukaryotic organisms. In mammals, at least seven CK1 isoforms (α, β, γ1, γ2, γ3, δ, and ε) have been identified [12]. In *S*. *cerevisiae*, four CK1 kinases (Yck1, Yck2, Yck3 and Hrr25) have been identified and all CK1 kinases except Hrr25 contain a C-terminal di-cysteine motif for palmitoylation, which mediates membrane association [13]. In *S*. *cerevisiae*, Hrr25 is involved in regulating diverse events including clathrin-mediated endocytosis [14], ER-to-Golgi traffic [15], DNA repair [16], and both selective autophagy and macroautophagy pathways [17,18]. In *Aspergillus nidulans*, the Hrr25 ortholog CkiA is required for CreA-mediated catabolite repression [19] and the trafficking of amino acid transporters to the plasma membrane [20]. Although Hrr25 is not essential for cell viability in budding yeast, the deletion of *HRR25* orthologues is lethal in some fungi including *A*. *nidulans*, *F. graminearum*, *Magnaporthe oryzae* [7,21,22]. Yeast Yck1 and Yck2 are involved in regulating cellular morphogenesis, cytokinesis, nutrient sensing, septin assembly, and endocytosis [23,24]. Both of them localize to the plasma membrane and have a C-terminal di-cysteine motif for palmitoylation, which mediates membrane association [13]. The Yck1 and Yck2 kinases also regulate the localization and activity of Mss4 that directs the synthesis of phosphatidylinositol 4,5-bisphosphate (PI4,5P_2_) at the plasma membrane [25]. In the white yeast *Candida albicans*, CaYck2 plays important roles in regulating yeast-hyphal transition and biofilm formation as well as being involved in both Mpk1 and Hog1 MAPK pathways [26]. Although there are only limited studies, the *YCK1/2* kinase genes are well conserved in filamentous ascomycetes. In the model filamentous fungus *Neurospora crassa*, the *YCK1/2* ortholog CK1a is assumed to be essential for growth [27] and it plays a critical role in temperature compensation of the circadian rhythm by phosphorylating the FRQ protein [28]. In the rice blast fungus *M. orzyzae*, the *Moyck1* deletion mutant is viable but defective in vegetative growth, appressorium formation, and plant infection. *MoYCK1* also regulates autophagy and responses to ionic hyperosmotic and heavy metal cation stresses [22].

Bni4 was originally identified as a scaffold protein that is involved in chitin biosynthesis and septation in *S. cerevisiae*. It tethers the chitin synthase catalytic subunit Chs3 to the bud neck by linking the chitin synthase regulatory subunit Chs4 to the septin Cdc10 [29,30]. Bni4 is also the targeting component of the Bni4-Glc7 type I phosphatase (PP1) to the bud neck [31]. The Glc7 PP1 is involved in cell polarity, cell wall integrity and morphology [32]. Moreover, several polarity genes including Spa2 and Bni1 interact with Bni4 by genetic analysis in *S. cerevisiae* [33]. In *C. albicans*, the *Cabni4* null mutant is reduced in cell wall chitin deposition and hyphal formation [34]. The exact function of Bni4 orthologs in filamentous fungi has not been characterized. Rho1, a small GTPase that regulates actin organization by interacting with Bni1 formin, is required for polarized growth and cell wall integrity in yeast and filamentous fungi. In *S. cerevisiae*, Rho1 activates the Pkc1-Slt2 MAP kinase cell wall integrity (CWI) pathway [35], acts as the regulatory subunit of β-1,3-glucan synthase [36,37], and is required for vacuole fusion [38,39]. In *N. crassa*, the conditional *rho-1* mutant forms swollen hyphal tips that can re-establish polarized growth at restrictive temperatures [37]. However, the relationship among CK1, Bni4 and Rho1 is still unclear.

Limited studies in *N. crassa*, *M. oryzae*, and *F. graminearum* have shown that the *YCK1*/2 orthologs are important for hyphal growth in filamentous fungi but their exact function is not clear [7,8,22,28]. In this study, we further determined the function of FgYck1 in polarized growth and cell wall integrity in *F. graminearum* and isolated and characterized spontaneous suppressors of the *Fgyck1* mutant. Our results show that *FgBNI4* and *FgYCK1* have stage-specific genetic relationships during vegetative growth and conidiation in *F. graminearum*, which may be related to FgRho1 signaling and conserved in other filamentous fungi for regulating polarized growth at hyphal tips and cell wall integrity.

## 2. Results

### 2.1. FgYCK1 Is Important for Vegetative Growth, Sexual/Asexual Development, and Pathogenesis

**Figure 1:**
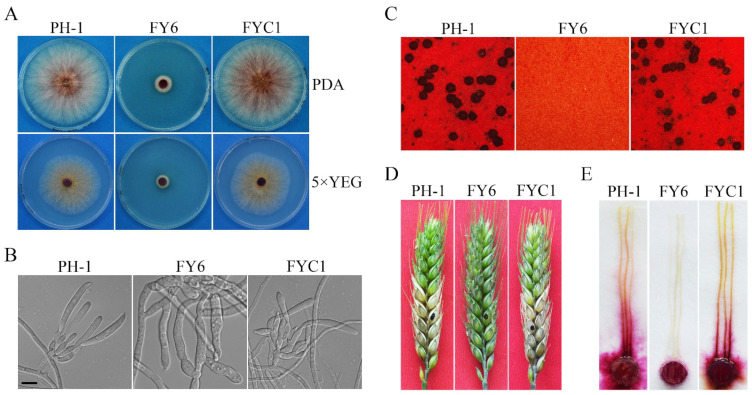
Defects of the *Fgyck1* mutant in vegetative growth, sexual/asexual reproduction and pathogenicity. (**A**) Three-day-old PDA and 5×YEG cultures of the wild-type PH-1, *Fgyck1* mutant (FY6) and *Fgyck1*/*FgYCK1* complemented transformant (FYC1). (**B**) Five-day-old CMC cultures of the same set of strains were examined for phialides and conidia. No conidia or phialides were observed in the *Fgyck1* mutant. Bar, 10 μm. (**C**) Mating cultures were examined at 7 days post-fertilization (dpf) for perithecium formation. (**D**) Flowering wheat heads inoculated with the indicated strains were examined for head blight symptoms at 14 days post-inoculation (dpi). Black dots mark the inoculated spikelets. (**E**) Corn silks inoculated with culture blocks were photographed at 5 dpi.

The *FgYCK1* (FGRRES_10066) gene encodes a 453-aa protein that contains a typical protein kinase domain (12-288 aa) and a C-terminal di-cysteine motif (Figure S1). It shares 69%, 70%, and 58% identity with yeast Yck1, Yck2, and Yck3, respectively. When grown on PDA, the *Fgyck1* deletion mutant (Table 1) formed compact colonies with limited aerial hyphae (Figure 1A) and grew at approximately 1.8 mm/day (Table 2), but the growth rate of the wild-type PH-1 was 11.3 mm/day. Microscopic examinations showed that the *Fgyck1* mutant was blocked in the production of phialides and conidia in CMC cultures, while the PH-1 produced abundant conidia (123.8 × 10^4^ conidia/mL) (Figure 1B; Table 2). On mating plates, the *Fgyck1* mutant failed to produce perithecia (Figure 1C). In infection assays with flowering wheat heads, the average disease index of *Fgyck1* mutant was 0, which is much lower than that (8.8) of PH-1, indicating that the *Fgyck1* mutant is non-pathogenic (Table 2; Figure 1D). The same result was also obtained in infection assays with corn silks (Figure 1E). When the full-length *FgYCK1* allele was re-introduced into the *Fgyck1* mutant, the resulting *Fgyck1*/*FgYCK1* transformant (Table 1) was similar to the wild type in vegetative growth, conidiation, sexual reproduction, and pathogenesis (Figure 1; Table 2). Therefore, deletion of *FgYCK1* is directly responsible for all the defects observed in the *Fgyck1* mutant.

### 2.2. Spontaneous Suppressors of Fgyck1 Are Partially Recovered in Growth Rate and Conidiation

**Figure 2:**
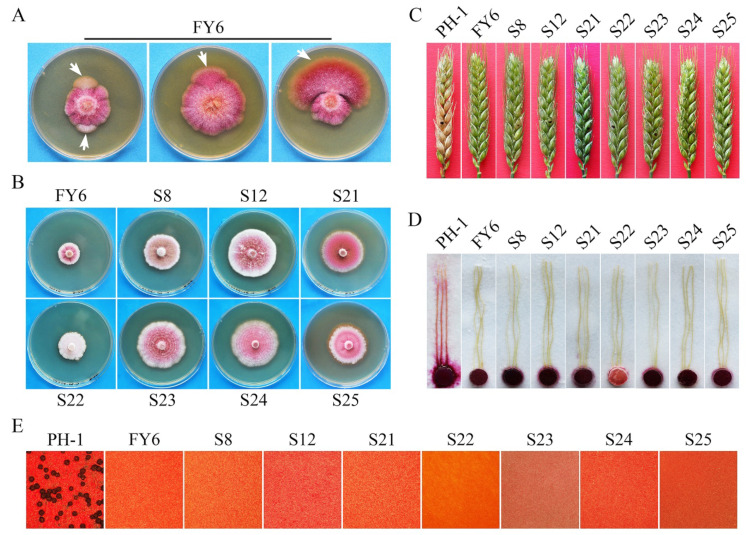
Spontaneous suppressors of the *Fgyck1* mutant. (**A**) Sectors with faster growth rate and different pigmentation (marked with arrows) were observed in PDA cultures of the *Fgyck1* mutant FY6 older than one week. (**B**) Five-day-old PDA cultures of the *Fgyck1* mutant and seven representative suppressors with different growth rate and pigmentation. (**C**) Flowering wheat heads inoculated marked strains were photographed at 14 dpi. Black dots indicate the inoculated spikelets. (**D**) Corn silks inoculated with culture blocks of the same set of strains were photographed at 5 dpi. (**E**) Mating cultures were examined for perithecium formation at 7 dpf. All suppressors were non-pathogenic and failed to produce perithecia.

The *Fgyck1* mutant was unstable and often produced fast-growing sectors in cultures older than 7 days (Figure 2A). A total of 25 spontaneous suppressors that grew over 18% faster than the original mutant were collected. These suppressor strains varied in growth rate and colony morphology as shown in Figure 2B with seven representative suppressor strains. Although they grew faster than the original *Fgyck1* mutant, all the suppressor strains still grew slower than the wild-type strain PH-1 (Table S1; Figure 2B). When assayed for conidiation in CMC, 15 of the 25 suppressors produced conidia but at a significantly reduced level in comparison with the wild type (Table S1). Furthermore, conidia produced by these suppressors were shorter and had fewer septa than those of PH-1 (Table S1; Figure S2). Like the *Fgyck1* mutant, all the 25 suppressor strains were nonpathogenic in infection assays with wheat heads (Figure 2C; Table S1) and corn silks (Figure 2D). They also were sterile and failed to produce perithecia on mating plates (Figure 2E). These results indicate that none of these suppressor strains were fully rescued in the defects of the *Fgyck1* mutant although its defects in vegetative growth and conidiation were partially suppressed.

### 2.3. Identification of Mutations in Spontaneous Suppressor Strains of Fgyck1

In *F. graminearum*, spontaneous suppressor mutations can be efficiently identified by whole-genome sequencing [42,43]. The seven representative suppressor strains (S8, S12, S21, S22, S23, S24, and S25) and original *Fgyck1* mutant were sequenced over 100× coverage by Illumina Hi-seq. In comparison with the genome sequence of the *Fgyck1* mutant, a total of 11 mutations were identified in eight predicted genes (Table 3). All these mutations were verified by PCR amplification and sequencing analysis of the corresponding genes. Two of these seven suppressor strains, S12 and S23, had mutations in FGRRES_07218 that are homologous to yeast *BNI4* (named *FgBNI4* in this study). Three suppressor strains, S21, S24, and S25, had the D80N mutation in FGRRES_03646 that is homologous to the *TNA1* high-affinity nicotinic acid permease gene in *S. cerevisiae* (named *FgTNA1*). For the other six genes, only a single suppressor mutation was identified in one of these seven suppressor strains (Table 3), including the nonsense, deletion, and missense mutations in genes orthologous to yeast *IES4*, *MIG1*, and *MSS4*, respectively.

**Figure 3:**
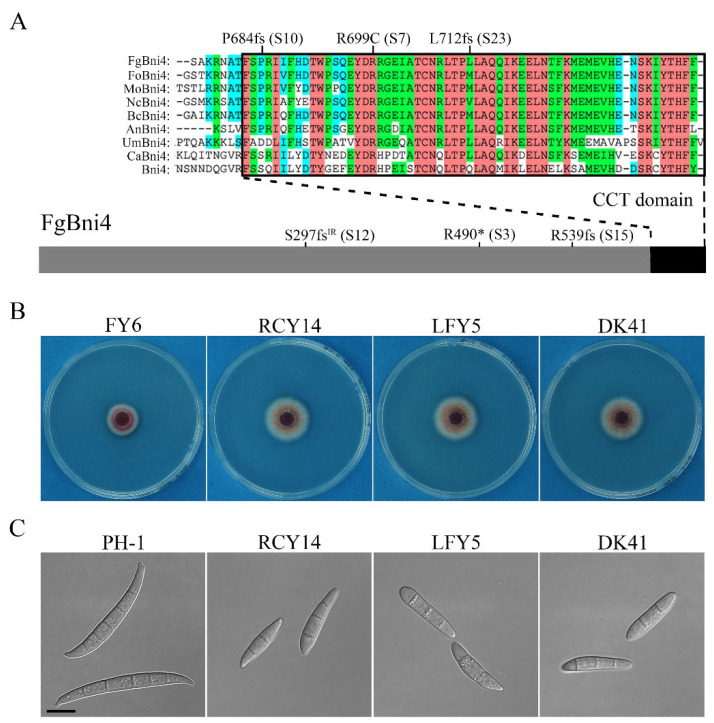
Suppressive mutations in *FgBNI4* and their effects on the *Fgyck1* mutant. (**A**) Schematic drawing of FgBni4 and positions of suppressor mutations. Sequence alignments of the conserved C-terminal region (black box) of FgBni4 with its orthologs from *Fusarium oxysporum* (Fo), *Magnaporthe oryzae* (Mo), *Neurospora crassa* (Nc), *Botrytis cinerea* (Bc), *Aspergillus nidulans* (An), *Ustilago maydis* (Um), *Candida albicans* (Ca), and *Saccharomyces cerevisiae* (Sc). The star (∗) indicates stop codon mutation. (**B**) Three-day-old PDA cultures of *Fgyck1* (FY6), *Fgyck1 FgBNI4*^R699C^ (RCY14), *Fgyck1 FgBNI4*^ΔCT^ (LFY5), and *Fgyck1 Fgbni4* (DK41) mutants. (**C**) Conidia of indicated strains harvested from 5-day-old CMC cultures. Bar, 10 μm.

Because spontaneous mutations in *FgBNI4* and *FgTNA1* were identified in more than one suppressor strain sequenced, we amplified and sequenced these two genes from the remaining 18 suppressor strains. Additional four suppressors were found to have mutations in the open reading frame (ORF) of *FgBNI4*. To our surprise, no additional suppressor strains had mutation in *FgTNA1* (Table S2). The six suppressor strains with mutations in *FgBNI4* differed from each other in genetic mutations (Table 3; Figure 3A). In suppressor S3, a nonsense mutation occurred at R490 (R490*), resulting in the truncation of 490-741 aa of the FgBni4 protein. Suppressor S12 had the G^889−1^T to AT mutation at the splicing site of the first intron, resulting in the retention of the 78-bp intron and a frame shift. Suppressors S10, S15, and S23 also had frame-shift mutations that were caused by deletion of CT^2051–2052^, AC^1617–1618^, and CT^2134–2135^, respectively. Interestingly, unlike the other five suppressors, S7 had one missense mutation that resulted in the R699 to C change in the FgBni4 protein.

Sequence alignment showed that the C-terminal region of FgBni4 (682–741 aa) is well conserved among its orthologs from other fungi (Figure S3) and named CCT (for conserved C-terminal tail) in this study. The R699C missense mutation in suppressor S7 occurred at a well-conserved R residue in this CCT region. Whereas the nonsense mutation at R490 and frameshift mutations caused by intron retention or deletion of AC^1617–1618^ resulted in the truncation of more than one third of FgBni4 protein, the CCT region was only partially truncated by ΔCT^2051–2052^ and ΔCT^2134–2135^ mutations in suppressors S10 and S23.

### 2.4. The R699C Mutation in FgBNI4 Is Verified for Its Suppressive Effect on Fgyck1

Because the *Fgyck1* mutant was unstable, to verify the suppressive effect of the R699C mutation we had to first generate the *FgBNI4*^R699C^ mutant. The R699C mutation was introduced into *FgBNI4* by overlapping PCR with primers carrying the C^2095^GC to T^2095^GC mutation (Table S4). The resulting PCR product was then used to generate the *FgBNI4*^R699C^ gene replacement construct (Figure S4A) with the *hph* hygromycin phosphotransferase as the selectable marker. After transformation of PH-1, transformants resistant to hygromycin were screened by PCR for the replacement of endogenous *FgBNI4* with *FgBNI4*^R699C^ (Figure S4A). The resulting *FgBNI4*^R699C^ mutants were further confirmed by PCR and sequencing analysis.

We then transformed the *FgYCK1* gene replacement construct carrying the *neo* marker into the *FgBNI4*^R699C^ mutant. Transformants resistant to both hygromycin and geneticin were screened for deletion of *FgYCK1* to identify the *Fgyck1 FgBNI4*^R699C^ mutants (Figure S5B). Like suppressor S7, the *Fgyck1 FgBNI4*^R699C^ mutant grew faster than the *Fgyck1* mutants but slower than PH-1 (Figure 3B). These produced conidia, although conidiation was significantly reduced compared to the wild type (Table 2). Furthermore, conidia formed by the *Fgyck1 FgBNI4*^R699C^ mutants were shorter and had fewer septa than the wild-type conidia (Figure 3C). In infection assays with wheat heads, the *Fgyck1 FgBNI4*^R699C^ mutants were nonpathogenic (Figure S6A). On selfing mating plates, the *Fgyck1 FgBNI4*^R699C^ mutants were sterile and failed to form perithecia (Figure S6B). These results confirm that the R699C mutation in *FgBNI4* partially suppresses the defects of *Fgyck1* in vegetative growth and conidiation but has no effect on its defects in pathogenesis (infectious growth) and sexual reproduction.

### 2.5. Deletion of Entire FgBNI4 and Truncation of Its CCT Region Have the Same Suppressive Effect on the Fgyck1 Mutant

Among all the suppressor strains with frameshift mutations, deletion of CT^2134–2135^ in S23 resulted in the shortest truncation of the CCT region of FgBni4 (truncation of 29 aa). To verify its suppressive effect on *Fgyck1*, we first generated the *FgBNI4*^ΔCT^ gene replacement construct (Figure S4B) and transformed it into PH-1. The *FgBNI4*^ΔCT^ mutant (Table 1) was verified for the deletion of CT^2134–2135^ by sequencing analysis (Figure S4B) and transformed with the *FgYCK1* gene replacement construct (Figure S5B). The resulting *Fgyck1 FgBNI4*^ΔCT^ double mutant (Table 1) was partially rescued in the defects of the *Fgyck1* mutant in growth (Table 2; Figure 3B) and produced few conidia with morphological defects (Figure 3C). However, like suppressor S23, the *Fgyck1 FgBNI4*^ΔCT^ mutant was nonpathogenic and sterile in selfing (Figure S6), indicating that truncation of the CCT region of *FgBNI4* also partially rescued the defects of *Fgyck1* mutant in growth and conidiation but had no effect on plant infection and perithecium development. Interestingly, all suppressor strains with mutations in *FgBNI4* (S3, S7, S10, S12, S15 and S23) were partially rescued in conidiation and had the same defect in conidium morphology (Table S1; Figure S2).

To further characterize the relationship between FgYck1 and FgBni4, we also generated the *Fgbni4* deletion mutant and transformed it with the *FgYCK1* gene replacement construct (Figure S5). The resulting *Fgbni4 Fgyck1* double mutant (Table 1) had similar phenotypes with the *Fgyck1 FgBNI4*^ΔCT^ and *Fgyck1 FgBNI4*^R699C^ mutants in growth, conidiation, sexual reproduction, and pathogenesis (Figure 3B,C; Figure S6). These results indicate that truncation of the C-terminal 29 aa residues had the same suppressive effects as deletion of the entire *FgBNI4* on the *Fgyck1* mutant. Therefore, the CCT region of *FgBNI4* must be essential for its function. Other nonsense and frameshift mutations that occurred upstream from CT^2134–2135^ likely had the same effects as deletion of *FgBNI4*.

### 2.6. FgBNI4 Is Involved in Cell Wall Integrity and Hyphal Growth

**Figure 4:**
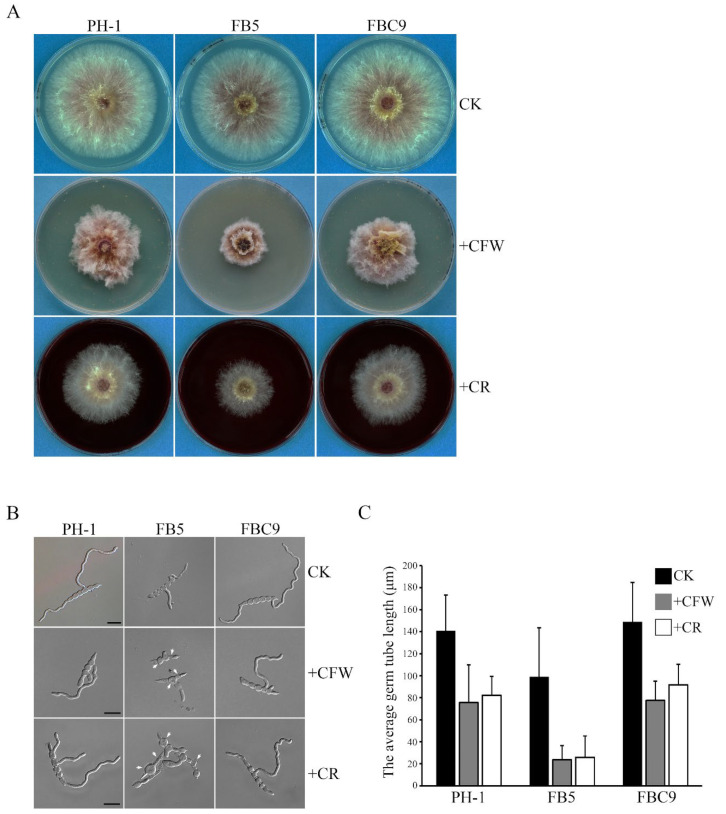
The involvement of FgBni4 in response to cell wall stresses in *F. graminearum*. (**A**) Colonies of wild-type strain (PH-1), *Fgbni4* mutant (FB5), and *Fgbni4*/*FgBNI4* complemented transformant (FBC9) grew on PDA plates supplemented without (CK) or with 200 μg/mL CFW or 300 μg/mL CR for 3 days. (**B**) Conidia of indicated strains were incubated in YEPD without (CK) or with 20 μg/mL CFW or 15 μg/mL CR for 6 h before examination. White arrows point to the swollen or ruptured cells of *Fgbni4* mutant. Bar, 20 μm. (**C**) Hyphae length of indicated strains without (CK) or with CFW or CR treatments. The length of hyphae was measured by using Image J software.

The *Fgbni4* mutant was slightly reduced in vegetative growth (Figure 4A; Table S3) but normal in asexual and sexual reproduction (Figure S7; Table S3). In infection assays with flowering wheat heads, the *Fgbni4* mutant caused typical FHB symptoms in the inoculated wheat kernels and was able to spread to nearby spikelets (Figure S7C). Nevertheless, its disease index was slightly lower than that of PH-1 (Table S3). These results indicate that *FgBNI4* is dispensable for asexual and sexual reproduction but plays a role in vegetative growth and plant infection.

In *S. cerevisiae* and *C. albicans*, Bni4 is involved in chitin deposition in cell wall [29,34]. To determine whether the *Fgbni4* mutant is defective in cell wall integrity, we first assayed the effects of cell wall disturbing compounds Calcofluor white (CFW) and Congo Red (CR) on hyphal growth. In the presence of 200 μg/mL CFW or 300 μg/mL CR, the *Fgbni4* mutant was more significantly reduced in growth rate than PH-1 (Figure 4A). We also assayed the effects of CFW and CR on conidium germination. After incubation in regular YEPD for 6 h, conidia of PH-1 and *Fgbni4* mutant had no obvious defects in germination, although germ tubes of *Fgbni4* mutant were shorter than those of PH-1 (Figure 4B). In the presence of 15 μg/mL CR, which had no obvious effect on PH-1, germination was not inhibited but germ tubes were much shorter in the *Fgbni4* mutant. Swollen conidium and hyphal compartments, as well as empty conidium compartments were often observed in the mutant but not in PH-1 (Figure 4B). The addition of 20 μg/mL CFW to YEPD had similar but more severe effects on germ tube growth and cell morphology in the *Fgbni4* mutant (Figure 4B,C). When the full-length *FgBNI4* gene was transformed into the *Fgbni4* mutant, the resulting transformants were normal in vegetative growth, pathogenicity, and responses to cell wall stressors (Figure 4). Therefore, *FgBNI4* is important for cell wall integrity, which may be directly related to its roles in vegetative growth and plant infection in *F. graminearum*.

### 2.7. The Fgyck1 Mutant Is Defective in Maintaining Polarized Growth

**Figure 5:**
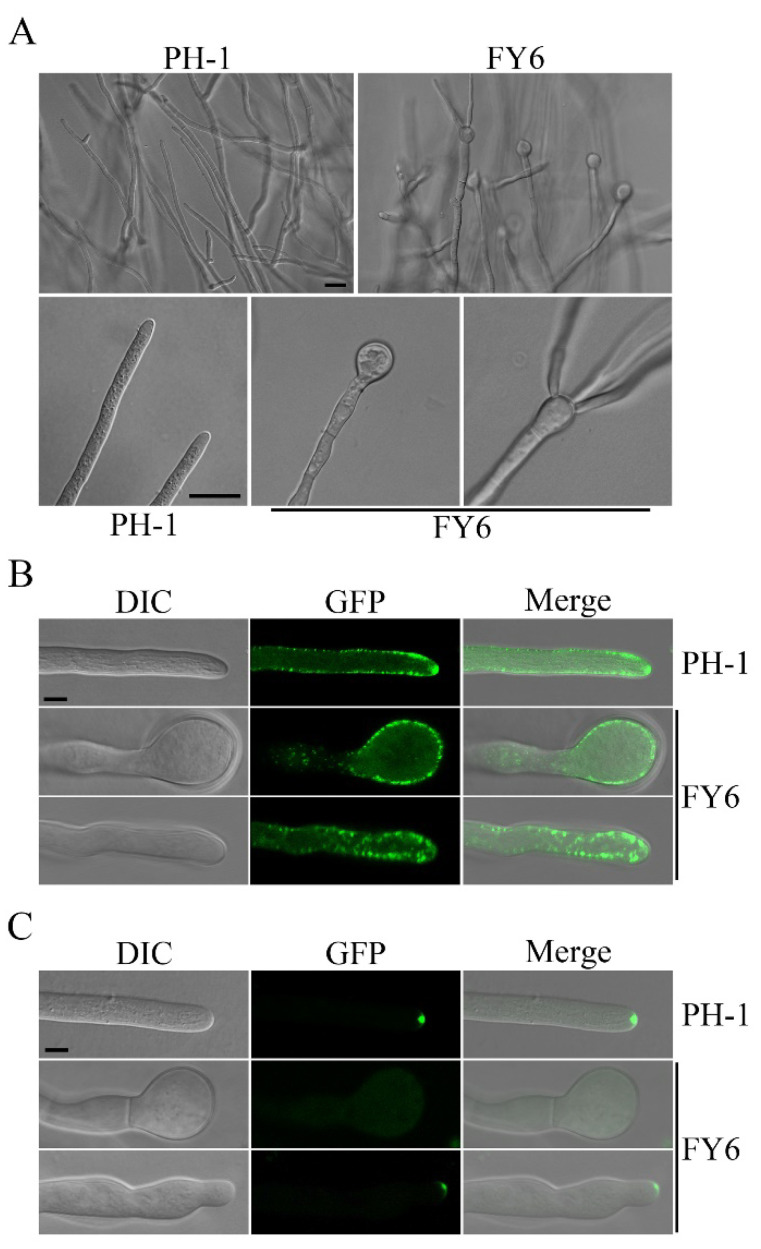
The *Fgyck1* mutant is defective in maintaining polarized hyphal growth. (**A**) Hyphae of PH-1 and *Fgyck1* mutant (FY6) from 36-h-old PDA cultures. Swollen hyphal tips were observed in the *Fgyck1* mutant and some of them produced one or multiple new tips or hyphae. Bar, 20 μm. (**B**) Hyphal tips of transformants of PH-1 and *Fgyck1* mutant FY6 expressing the LifeAct-GFP construct were examined by confocal microscopy. The enrichment of LifeAct-GFP at the core of Spk is not observed in normal or swollen hyphal tips of the *Fgyck1* mutant. Bar, 5 μm. (**C**) Hyphal tips of transformants of PH-1 and FY6 expressing the FgSpa2-GFP construct were examined by confocal microscopy. Localization of FgSpa2-GFP to the polarisome was absent in swollen tips and reduced in normal tips in the *Fgyck1* mutant. Bar, 5 μm.

To determine whether deletion of *FgYCK1* affects polarized growth, we examined hyphal tips after incubation for 36 h on PDA. Whereas all the wild-type hyphae examined had normal hyphal tips, approximately 70% of the hyphae had swollen tips in the *Fgyck1* mutant (Figure 5A). For some of these swollen hyphal tips, one or multiple new hyphae emerged and grew (Figure 5A), indicating the re-establishment of polarized growth. Therefore, FgYck1 likely plays an important role in maintaining polarized growth at hyphal tips in *F. graminearum*.

In filamentous fungi, defects or disturbances in actin assembly often lead to swollen hyphal tips [44,45]. To verify the role of FgYck1 in polarized growth, we transformed the F-actin marker LifeAct-GFP into PH-1 and the *Fgyck1* mutant. In the wild type, LifeAct-GFP signals were mainly observed in the subapical collar region of hyphae and enriched at the core of Spitzenkörper (Spk) bodies at hyphal tips (Figure 5B). However, in the *Fgyck1* mutant, LifeAct-GFP signals were observed in the peripherals of swollen hyphal tips and no strong LifeAct-GFP signals at the Spk core were observed when more than 50 swollen hyphal tips were examined (Figure 5B). We also examined more than 50 hyphae of the *Fgyck1* mutant with relatively normal hyphal tips and enhanced LifeAct-GFP signals at the Spk core were not observed (Figure 5B). These data indicate that the deletion of the *FgYCK1* gene affects the localization and enrichment of F-actin to the Spk1 core.

The polarisome complex mediates F-actin polarization and directs Spk vesicles to the hyphal apex [46]. FgSpa2 is the key component of the polarisome that is required for establishment and maintenance of polarized growth in *F. graminearum* and other fungi [47,48,49]. Therefore, we also transformed *FgSPA2*-GFP into PH-1 and the *Fgyck1* mutant. As expected, FgSpa2-GFP showed a polarisome-like localization at all hyphal tips in the wild type (Figure 5C). However, when we examined more than 50 swollen hyphal tips of the *Fgyck1* mutant, no FgSpa2-GFP signals were observed (Figure 5C). Although FgSpa2-GFP signals were present at the tip of normal hyphae of the *Fgyck1* mutant, its polarisome-like accumulation was significantly impaired (Figure 5C). These results indicate that the loss of FgYck1 may affect the organization of the Spk body and polarisomes that are necessary to maintain polarized growth at the tip.

### 2.8. The Defect of Fgyck1 in Polarized Growth Is Alleviated by Deletion of FgBNI4

**Figure 6:**
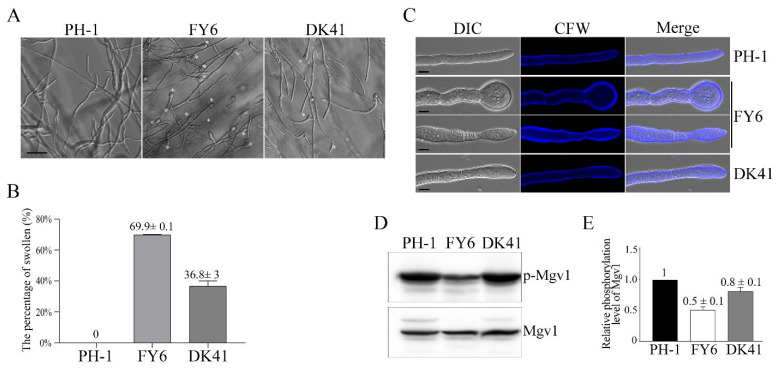
Deletion of *FgBNI4* partially suppresses the defects of *Fgyck1* mutant in polarized growth and cell wall integrity. (**A**) Hyphae of 36-h-old PDA cultures of the wild type (PH-1) and the *Fgyck1* (FY6) and *Fgyck1 Fgbni4* (DK41) mutants were examined for swollen tips (marked with asterisks). Bar, 50 μm. (**B**) The percentage of hyphae with swollen tips in PH-1, FY6, and DK41. (**C**) Hyphae of the marked strains collected from 12 h YEPD cultures were stained with CFW and observed by confocal microscopy. Bar, 5 μm. (**D**) Total proteins isolated from 18 h vegetative hyphae were used for Western blot analyses with the anti-TpEY and anti-Mgv1 antibodies. (**E**) The relative phosphorylation level of Mgv1 in the *Fgyck1* (FY6) and *Fgyck1 Fgbni4* (DK41) mutants in comparison with PH-1 (arbitrarily set to 1). The intensities of bands detected on Western blots were analyzed with the Image Lab^TM^ Software 3.0 (Bio-Rad Laboratories, Hercules, CA, USA). For each strain, the phosphorylation level of Mgv1 was estimated by comparing the intensities of the band detected with the anti-TpEY antibody with the band detected with the anti-Mgv1 antibody.

When hyphal tips were examined, the *Fgyck1 Fgbni4* mutant still formed a few apical swollen bodies (Figure 6A). However, in comparison with the *Fgyck1* mutant, the percentage of swollen hyphal tips was significantly reduced in the double mutant. Whereas about 70% of the hyphae had swollen tips in the *Fgyck1* mutant, over 63% of hyphal tips were normal in the *Fgyck1 Fgbni4* mutant (Figure 6B). When stained with CFW, irregular hyphal width and CFW staining were often observed in the *Fgyck1* mutant but rare in the *Fgyck1 Fgbni4* mutant (Figure 6C). Therefore, deletion of *FgBNI4* significantly alleviates the polarized growth defect of the *Fgyck1* mutant.

The irregular CFW staining in *Fgyck1* indicates the alteration of cell wall composition (Figure 6C), which may be associated with the CWI signaling pathway. In *F. graminearum*, the Mgv1 MAPK regulates CWI pathway and the *mgv1* deletion mutant also has severe growth defects [50]. Tip swelling observed in the *Fgyck1* mutant may be related to defects in CWI signaling. To determine whether deletion of *FgYCK1* affects the activation of the Mgv1 MAPK, we assayed its phosphorylation with a commercially available anti-TpEY phosphorylation-specific antibody. On Western blots with total proteins isolated from hyphae collected from 18 h YEPD cultures, the phosphorylation level of Mgv1 was reduced in the *Fgyck1* mutant in comparison with the wild type (Figure 6D,E). However, the expression level of Mgv1 detected with an anti-Mgv1 antibody generated in this study was not affected by deletion of *FgYCK1*. In the *Fgyck1 Fgbni4* double mutant, Mgv1 phosphorylation was recovered to the wild-type level (Figure 6D,E), indicating that deletion of *FgBNI4* rescues the defect of *Fgyck1* in Mgv1 activation.

### 2.9. Deletion of FgBNI4 Alleviates the Defects of Fgyck1 in FgRho1 Internalization and Vacuolar Fusion

**Figure 7:**
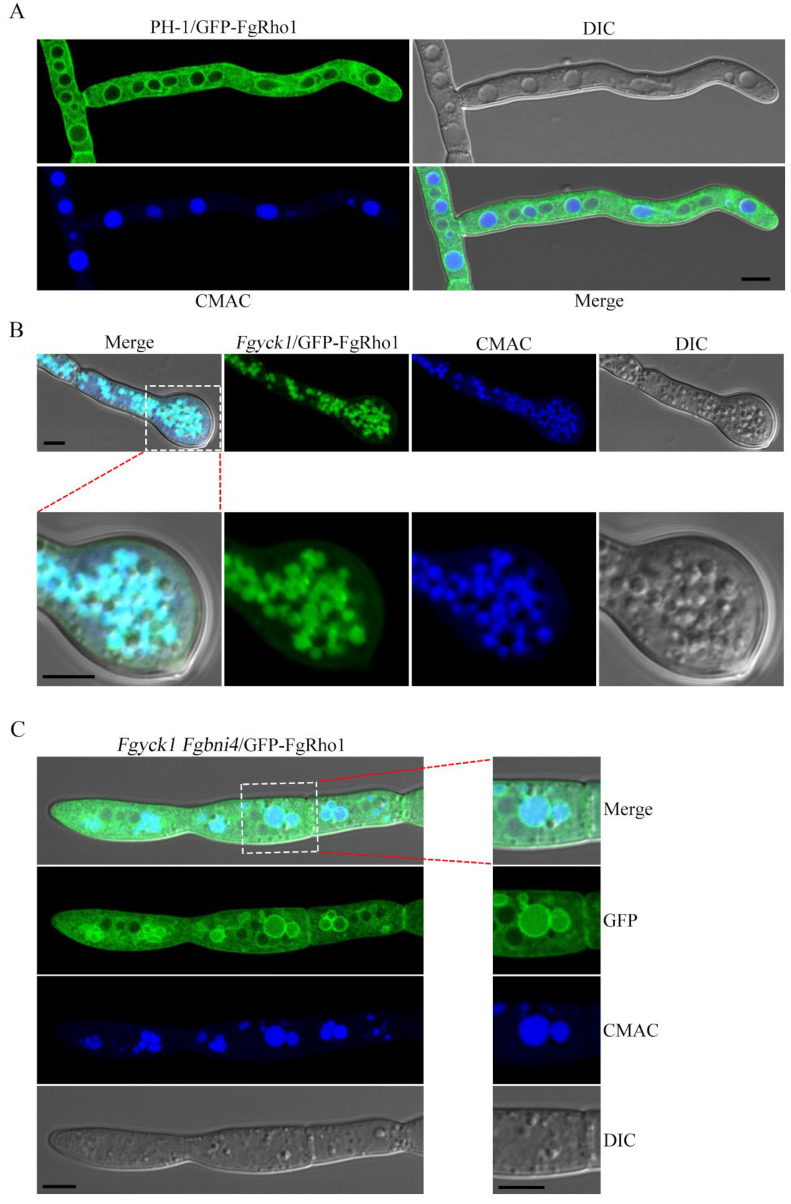
Localization of GFP-FgRho1 in the wild type, *Fgyck1*, and *Fgyck1 Fgbni4* strains. (**A**) Hyphae of GFP-*FgRHO1* transformant of the wild-type strain PH-1. (**B**) Representative germ tubes of *Fgyck1* mutant expressing GFP-*FgRHO1*. The lower panels are close-up views of the swollen tip as marked. (**C**) Hyphae of *Fgyck1 Fgbni4*/GFP-*FgRHO1* transformant. The right panels are close-up views of the framed sections on the left. For all the strains, hyphae were harvested from 12 h YEPD cultures and examined by confocal microscopy. The CMAC dye was used to stain the vacuolar lumen. Bar, 5 μm.

Rho1 is a small GTPase that plays essential roles in polarized growth, actin organization, and cell wall integrity in fungi [51,52]. The *rho1* mutants of *N. crassa* and *F. oxysporum* have similar defects with the *Fgyck1* mutant, including swollen tips and altered cell wall composition [37,53]. Therefore, we speculated that deletion of *FgYCK1* may negatively impact FgRho1 localization and function. To test this hypothesis, we transformed the GFP-*FgRHO1* fusion construct into PH-1 and the *Fgyck1* and *Fgyck1 Fgbni4* mutants and observed the FgRho1 localization in 12 h hyphae. In all hyphae of wild type, the GFP-FgRho1 predominantly localized to the plasma membrane and vacuolar membrane (Figure 7A). In contrast, stronger GFP-FgRho1 signals were accumulated inside the vacuoles in >75% hyphae of the *Fgyck1* mutant (Figure 7B), indicating an enhanced internalization of FgRho1. However, the internalization of FgRho1 was observed in <10% hyphae of the *Fgyck1 Fgbni4* double mutant (Figure 7C). Furthermore, the *Fgyck1* mutant appeared to have more and smaller vacuoles than the wild type (Figure 7), which may be related to defect in vacuole fusion caused by the internalization of FgRho1. The abundance and size of vacuoles were similar between PH-1 and the *Fgyck1 Fgbni4* double mutant (Figure 7). These results indicate that deletion of *FgYCK1* negatively impacts the localization and function of FgRho1 in *F. graminearum*, which may contribute the pleiotropic defects of the *Fgyck1* mutant due to the importance of Rho1 in hyphal tip growth and cell wall integrity [36,37]. Deletion of *FgBNI4* alleviates the defects of *Fgyck1* in FgRho1 internalization and vacuole fusion, which may contribute to the recoveries of *Fgyck1 Fgbni4* mutant in polarized growth and cell wall integrity.

### 2.10. The FgYck1 Localizes to Plasma Membrane and Vacuolar Lumen

**Figure 8:**
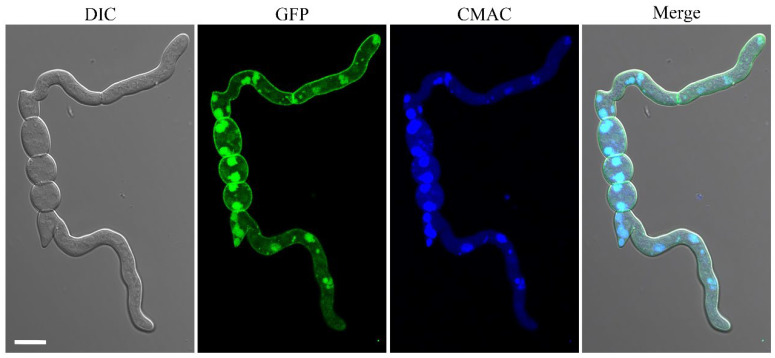
Subcellular localization of GFP-FgYck1. Hyphae of PH-1/GFP-*FgYCK1* transformant from 12-h-old YEPD cultures were examined by confocal microscopy. The GFP signals mainly localized to plasma membrane and vacuolar lumen. The CMAC dye was used to stain the vacuolar lumen. Bar, 10 μm.

To confirm the subcellular localization of FgYck1, we generated the GFP-*FgYCK1* under the control of the strong constitutive RP27 promoter [54] and expressed it in PH-1. In the 12 h hyphae of PH-1/GFP-*FgYCK1* transformant, GFP-FgYck1 signals were detected mainly at the plasma membrane and vacuolar lumens that were stained with CMAC (7-amino-4-chloromethylcoumarin, Sigma-Aldrich, Eugene, OR, USA), and weaker GFP signals were also observed in the cytoplasm (Figure 8).

## 3. Discussion

Whereas *S. cerevisiae* has two paralogous casein kinase I genes *YCK1* and *YCK2* originated from recent whole-genome duplication, *FgYCK1* is the only *YCK1/2* ortholog in *F. graminearum*. Unlike the yeast *yck1 yck2* double mutant that is inviable, the *Fgyck1* deletion mutant was viable but had severe growth defects. However, the *Fgyck1* mutant was blocked in conidiation. Even after incubation in CMC medium for over two weeks, no conidia were observed in mutant cultures. The production of conidia from phialides in *F. graminearum* is a similar blastospore formation process as the budding of yeast cells. Therefore, the *YCK1* orthologs may have a conserved role in blastospore formation on phialides. In yeast, *YCK1* and *YCK2* are important for proper septin assembly and morphogenesis. In *M. oryzae*, the *Moyck1* mutant is reduced in conidiation but still produces macroconidia [55], which are directly formed on conidiophores instead of phialides. However, microconidia are produced from phialides in *M. oryzae* [55] and the *Moyck1* mutant may be blocked in the production of microconidia.

Like the *Fgprp4*, *fng1*, and a few other *F. graminearum* mutants with severe growth defects [42,56,57,58], the *Fgyck1* mutant produced spontaneous suppressors with faster growth rates. By whole genome sequencing of seven selected suppressors, we identified mutations in seven predicted genes, including orthologs of yeast *BNI4*, *TNA1*, *MIG1*, and *MSS4*. Three of these had the same D80N mutation in *FgTNA1*, suggesting that this is a hot spot for spontaneous mutations. However, no additional mutation in *FgTNA1* was identified in the remaining 18 suppressor strains by amplification and sequencing of its coding region. Interestingly, all three of the suppressors with the D80N mutation in *FgTNA1* also had mutations in other genes. Therefore, we did not pursue further characterization of *FgTNA1* in this study. One missense suppressor mutation was identified in the ortholog of *A. nidulans CREA* and *S. cerevisiae MIG1*. The Mig1/CreA protein is a major regulator of carbon catabolite repression in yeast and filamentous fungi [59,60]. Because Yck1 and Yck2 are involved in glucose sensing and signaling in yeast [61], it is likely that FgYck1 also is involved in the utilization of different carbon sources in *F. graminearum*, possibly via FgCreA.

*FgBNI4* was selected for further characterization because it is the only other gene with mutations identified in more than one suppressor strain by whole-genome sequencing [32,33,34,35,36,37]. In *F. graminearum*, the *Fgbni4* deletion mutant was normal in conidiation and sexual reproduction but was reduced in growth rate and virulence. Interestingly, five out of the six suppressor strains with mutations in *FgBNI4* had nonsense or frameshift mutations that resulted in the truncation of its C-terminal region. The amino acid sequences of the FgBni4 orthologs are not well conserved among different fungi except the C-terminal 60 residues (named CCT region). The only missense mutation in *FgBNI4* that occurred in this region in suppressor S7 is at R699, a well-conserved residue among its orthologs. In addition, the frameshift mutation in suppressor S23 (ΔCT^2134–2135^, L712fs) resulted in the truncation of only the last 29 residues. Furthermore, both the R699C mutation and deletion of CT^2134–2135^ had the same suppressive effects with deletion of the entire *FgBNI4* gene on *Fgyck1* mutant in vegetative growth and conidiation. Therefore, this CCT region must be essential for the function of *FgBNI4*. Because deletion of *FgBNI4* and all the suppressor mutations in *FgBNI4* failed to suppress the defects of *Fgyck1* in sexual reproduction and plant proteins, we conclude that the genetic relationship between *FgBNI4* and *FgYCK1* is stage-specific during vegetative growth and asexual reproduction.

In *S. cerevisiae*, *YCK1* and *YCK2* have not been reported to physically or genetically interact with *BNI4*. In *F. graminearum*, mutations in the CCT region of *FgBNI4* partially suppressed the growth defect of the *Fgyck1* mutant. This genetic relationship between *FgYCK1* and *FgBNI4* may be related to their direct interaction and FgBni4 may be a target of FgYck1 kinase. Because Bni4 is a protein that mainly functions to interact with other proteins for their proper localization in yeast, it is possible that phosphorylation of FgBni4 by *Fgyck1* is necessary for its interaction with some FgBni4-interacting proteins in *F. graminearum*. However, because Bni4 is a subunit of the Bni4-Glc7 phosphatase I complex, it is also possible that suppressive effects of mutations in *FgBNI4* on *Fgyck1* are related to the antagonistic effects of phosphorylation by FgYck1 and dephosphorylation by Glc7 on their common targets. Disruption of *FgBNI4* will reduce or eliminate the dephosphorylation of FgYck1-phosphorylated proteins by the Bni4-Glc7 phosphatase I. Although Bni4 is not known to be phosphorylated by Yck1 or Yck2 in yeast, it is phosphorylated in a cell cycle-dependent manner by unknown kinases [62]. Yeast Bni4 is also phosphorylated by Slt2 and Kss1 MAP kinases that are orthologous to Mgv1 and Gpmk1, respectively [63]. In *F. graminearum*, it is possible that Mgv1 and Gpmk1 MAP kinases are also involved the phosphorylation of FgBni4. The *mgv1* mutant had severe growth defects although the *Gpmk1* mutant was only slightly reduced in growth. The *Gpmk1 mgv1* double has not been reported but the *Gpmk1 mgv1 Fghog1* triple mutant had more severe defects than the *mgv1* mutant [64].

The majority of the vegetative hyphae of the *Fgyck1* mutant had swollen tips [7,8]. However, conidium germination was not blocked and swollen tips of the *Fgyck1* mutant often re-established polarized growth and produced one or more new hyphae. Therefore, although *FgYCK1* is important for polarized growth, it is not essential for establishing polarized tip growth. Swollen hyphal tips observed in the *Fgyck1* mutant had only dispersed, peripheral LifeAct-GFP signals, which is similar to the effects of inhibiting actin polymerization. The central actin core of Spk observed in hyphal tips of PH-1 was not observed in these swollen hyphal tips of the *Fgyck1* mutant. Even in *Fgyck1* hyphae with relatively morphologically normal tips, the enrichment of F-actin at the center of Spk1 was not observed. Consistent with these observations, the apical localization of polarisome scaffold protein FgSpa2 was not observed at swollen hyphal tips in the *Fgyck1* mutant. Polarisome is a complex of proteins that nucleates actin cables for polarized cell growth in the budding yeast and filamentous fungi [65]. It is possible that FgYck1 regulates polarized growth by phosphorylating certain components of the polarisome. Deletion of *FgBNI4* may negatively impact the interaction of Glc7 phosphatase I with the polarisome components that are phosphorylated by FgYck1 and their dephosphorylation, leading to a faster growth rate in the *Fgyck1 Fgbni4* double mutant. In *S. cerevisiae*, some polarisome-related genes, including *SPA2* and *BNI1*, have been found to interact with *BNI4* in a synthetic genetic array analysis [33].

In *S. cerevisiae*, mutations in *BNI4* affect the localization of the chitin synthase III and chitin synthesis at the bud neck [31]. It is possible that deletion of *FgYCK1* also affects the expression or localization of chitin synthases in *F. graminearum*. Similar to the *Fgyck1* mutant, the *Gzchs5* and *Gzchs7* mutants produce swollen hyphal tips in *F. graminearum* [66,67]. In *C. albicans*, the *yck2* mutant is defective in cell wall integrity but has increased compensatory chitin deposition in the cell wall by up-regulating chitin synthase genes [26]. We noticed that the *Fgyck1* mutant had a stronger CFW staining of the cell wall than the wild type and CFW staining became normal in the *Fgyck1 Fgbni4* double mutant. Therefore, it is possible that deletion of *FgYCK1* also results in a thickening of the cell wall by up-regulating GzChs5, GzChs6, and other enzymes involved in cell wall synthesis. Consistent with this hypothesis, we found that the *Fgyck1* mutant was reduced in the phosphorylation of Mgv1 CWI MAP kinase [50] and deletion of *FgBNI4* rescued its Mgv1 phosphorylation to the wild-type level.

In *F. graminearum*, deletion of *FgYCK1* enhanced the internalization of FgRho1 into vacuoles, which was suppressed by deletion of *FgBNI4*. The vacuole fusion defect of the *Fgyck1* mutant was also partially rescued in the *Fgyck1 Fgbni4* double mutant. Therefore, it is likely that the defects of the *Fgyck1* mutant and suppressive effects of mutations in *FgBNI4* are related to their functional relationships with FgRho1 in *F. graminearum*. In yeast, Yck1 and Yck2 regulate the localization and activity of Mss4 for the synthesis of PI4,5P_2_ that is required for clathrin-mediated endocytosis [25,68] and involved in actin cytoskeleton organization and cell wall integrity through Rho1 signaling [25,69]. Interestingly, suppressor S12 had the N353T mutation in *FgMSS4*, which may partially suppress the defect of *Fgyck1* mutant in the localization or activity of FgMss4. FgYck1 may regulate the internalization of FgRho1 by controlling PI4,5P_2_ synthesis via phosphorylation of FgMss4 in *F. graminearum*. Therefore, it is important to characterize the functional relationship among the well-conserved FgYck1 kinase, FgRho1, and FgBni4 in hyphal growth, PI4,5P_2_-dependent endocytosis, and cell wall integrity in future studies.

In summary, our study showed that the *Fgyck1* mutant displayed pleiotropic defects in vegetative growth, conidiation, sexual development and pathogenicity. Deletion of *FgYCK1* also affected polarized growth and cell wall integrity. Through characterizing natural suppressor mutants, we found a novel genetic interaction between FgYck1 and FgBni4 during vegetative growth and conidiation. Additionally, the loss of *FgBNI4* can partially restore the phosphorylation of Mgv1 and correct the localization of FgRho1 in *Fgyck1* mutant. Further characterization of FgYck1 and FgBni4 on PI4,5P_2_ synthesis and endocytosis on the plasma membrane will be helpful in defining and clarifying the relationship between FgYck1 and FgBni4 in *F. graminearum*.

## 4. Materials and Methods

Fungal strains and culture conditions: The wild-type strain PH-1 and its mutants listed in Table 1 were routinely cultured on PDA (20% potato, 2% glucose, and 1.5% agar) at 25 °C and preserved in 20% glycerol at −80 °C. Conidiation was examined with liquid CMC (1.5% carboxymethylcellulose, 0.1% NH_4_NO_3_, 0.0% MgSO_4_·7H_2_O, 0.1% yeast extract) cultures [70]. Mating on carrot agar plates and examination for perithecium formation and ascospore ejection were assayed as described [71]. Protoplast preparation and polyethylene glycol (PEG)-mediated transformation were performed as described [50,72]. For transformant selection, hygromycin B (CalBiochem, La Jolla, CA, USA), geneticin (Sigma, St. Louis, MO, USA), and zeocin (Invitrogen, Carlsbad, CA, USA) were added to the final concentration of 300, 400 and 700 μg/mL, respectively, to both the bottom and top agar. Fungal genomic DNA was extracted from mycelia harvested from 24 YEPD cultures by the cetyltrimethylammonium bromide (CTAB) protocol as described [73].

Infection assays: Conidia of PH-1 and mutant strains were harvested from 5-day-old CMC cultures and resuspended to a concentration of 2 × 10^5^ conidia/mL in sterile distilled water. For wheat head infection assays, the fifth floret from the bottom of each head of cultivar Xiaoyan 22 was inoculated with 10 μL of conidial suspension or culture blocks [50,74]. Inoculated wheat heads were covered with a plastic bag to keep humidity for 48 h. Infected wheat plants were examined at 14 days post-inoculation (dpi) to estimate the disease index [50]. Infection assays with corn silks were carried out as described [75].

Isolation of spontaneous suppressors and identification of suppressor mutations: Sectors with faster growth rate were isolated as spontaneous suppressors from the edge of *Fgyck1* colonies formed on PDA plates as described [42,56,57]. To identify mutations in seven representative suppressor strains, genomic DNA samples were sequenced with the Illumina HiSeq-PE150 at Novogene Bioinformatics Institute (Beijing, China) to 50×coverage with pair-end libraries [76,77]. The resulting sequence reads were mapped onto the genome sequences of PH-1 and original *Fgyck1* mutant as a reference using Bowtie 2 [78]. Mutations were identified by SAMtools with the default parameters [79]. Annotation of the mutation sites was performed with the Variant Effect Predictor (VEP) program [80]. To determine whether the remaining 18 suppressors of *Fgyck1* have mutations in *FgBNI4* and *FgTNA1*, PCR products amplified with primer pairs FgBNI4/TF-FgBNI4/TR and FgTNA1/TF-FgTNA1/TR (Table S4) were sequenced at Sango Biotech (Shanghai, China).

Generation of the *Fgbni4* and *Fgbni4 Fgyck1* mutants: To generate the *Fgbni4* mutant, a 1.2-kb upstream and a 1.1-kb downstream flanking fragments of the *FgBNI4* gene were amplified with primer pairs B1F-B2R and B3F-B4R (Table S4), respectively, and fused to the *hph* fragments amplified with primer pairs HT/F-HY/R and YG/F-HT/R from pCB1003 by overlapping PCR [81,82]. The resulting PCR products were co-transformed into protoplasts of PH-1. Hygromycin-resistant transformants were screened for *FgBNI4* deletion by PCR with primer pairs H850-H852, B5F-B6R, B7F-H855R, and H866F-B8R. To generate the *Fgyck1 Fgbni4* double mutant, the *FgYCK1* replacement construct was generated with neomycin resistant cassette (*neo*) and transformed into the *Fgbni4* mutant. Transformants resistant to both hygromycin and geneticin were screened by PCR with primer pairs G850-G852, Y5F-Y6R, Y7F-G855R, and G866F-Y8R (Table S4) for the deletion of *FgYCK1*. Optimal conditions for the PCR reactions were as follows: 5 min denaturation at 95 °C followed by 35 cycles of 95 °C for 1 min, 57 °C for 1 min, and 72 °C for 1 min. The reaction was completed with a 10-min extension at 72 °C. All PCR reactions were performed in a C1000 Touch Thermal Cycler (Bio-Rad, Munich, Germany).

Generation of the *FgBNI4*^R699C^ *Fgyck1* and *FgBNI4*^ΔCT^ *Fgyck1* mutants: A modified split marker approach [81] was used to generate the transformants with in situ mutations. A DNA fragment containing the last 1000-bp of *FgBNI4* ORF and 400-bp terminator sequence was amplified by overlapping PCR [83] to introduce the R699C mutation with primer pairs P1F-P1R and P2F-P2R. The 800-bp DNA sequence downstream from the terminator sequence of *FgBNI4* was also amplified. These two fragments were ligated to the *hph* fragments amplified with primer pairs HT/F-HY/R and YG/F-HT/R by overlapping PCR and transformed into protoplasts of PH-1. The resulting *FgBNI4*^R699C^ mutants were confirmed by sequencing analyses. The *FgYCK1* replacement construct (*neo*) was then transformed into the *FgBNI4*^R699C^ mutant to generate the *FgBNI4*^R699C^ *Fgyck1* mutants. The same approach was used to generate the *FgBNI4*^ΔCT^ and *FgBNI4*^ΔCT^ *Fgyck1* mutants. All the primers are listed in the Table S4.

Complementation assays with the *Fgyck1* and *Fgbni4* mutants: For complementation of the *Fgyck1* mutant, the full-length *FgYCK1* gene, including the 2.3-kb promotor and 1-kb terminator regions, was amplified and cloned into vector pFL7 (carrying geneticin resistance marker) by the gap repair approach [84]. The resulting construct was confirmed by sequencing analysis and transformed into the *Fgyck1* mutant. For complementation of the *Fgbni4* mutant, the entire *FgBNI4* gene, with its promotor and terminator regions, was amplified and cloned into the GFP-tagged vector pKNTG [85] that carries the *neo* selectable marker by using the NovoRec^®^ plus One step PCR Cloning kit (Novoprotein, Shanghai, China). The resulting construct was transformed into the *Fgbni4* mutant. Both *Fgyck1*/*FgYCK1* and *Fgbni4*/*FgBNI4* transformants were confirmed by PCR analysis and assayed for phenotype changes.

Assays for defects in responses to cell wall stresses: The final concentration of 300 μg/mL Congo red (CR) and 200 μg/mL Calcofluor white (CFW) were added to PDA to assay vegetative growth at 25 °C as described [86,87]. Colony morphology was examined and photographed after incubation for 3, 5, or 7 days. To assay conidial germination, 20 μg/mL CFW or 15 μg/mL CR was added to freshly harvested conidia resuspended to 10^6^ conidia/mL in YEPD and incubated for 6 h at 25 °C [88]. All experiments were repeated independently at least three times.

Generation of the GFP-*FgRHO1*, GFP-*FgYCK1*, and *FgSPA2*-GFP transformants: All GFP fusion constructs were generated using the NovoRec^®^ plus One step PCR Cloning kit (Novoprotein, Shanghai, China). To generate the GFP-*FgRHO1* construct, the entire *FgRHO1* gene, including its terminator region, was amplified with primers GFP-RHO1/F and GFP-RHO1/R and fused with GFP in vector pKNTG-RP27, which was generated by inserting the constitutive RP27 promoter into pKNTG. The same approach was performed to generate the GFP-*FgYCK1* construct. To generate the *FgSPA2*-GFP construct, the entire coding region of *FgSPA2*, including its native promoter, was amplified with primers FgSPA2/F and FgSPA2/R and cloned into the *Kpn*I/*Hind*III-digested pKNTG. All resulting GFP fusion constructs were confirmed by sequencing analysis and transformed into protoplasts of PH-1 and the *Fgyck1* mutant.

Western blot analysis: Total proteins were isolated from hyphae harvested from 18 h YEPD cultures [88]. The Bradford method was performed to confirm the protein concentration [89] and 10 µg of protein were loaded per lane for each samples. Proteins separated on 10% SDS-PAGE gel were transferred to nitrocellulose membranes [90]. Phosphorylation of Mgv1 was detected with the PhophoPlus p44/42 MAP kinase antibody kit (Cell Signaling Technology, Danvers, MA, USA) as described [90]. To detect the expression level of Mgv1, an anti-Mgv1 antibody was generated at the ABclonal Biotechnology (Wuhan, China) by injecting rabbits with a polypeptide of Mgv1 (333–349 aa). On Western blots, a 46-kD Mgv1 band was detected in PH-1 but not in the *mgv1* mutant [50] with 1:5000 dilution of the resulting anti-Mgv1 antibody, indicating that it is suitable for Mgv1 detection. The Clarity^TM^ western ECL substrate kit (Bio-Rad, Hercules, CA, USA) and ChemiDoc^TM^ XRS+ imaging system (Bio-Rad, Hercules, CA, USA) were used for Western blot analysis. The Image Lab^TM^ software was used to analyze the quantitative changes of the Mgv1 phosphorylation level. Each experiment was repeated at least three times.

Vacuole and cell wall staining: Hyphae harvested from 12 h YEPD cultures were stained with 10 μM CMAC (7-amino-4-chloromethylcoumarin, Sigma-Aldrich) as described [91] to visualize the vacuolar lumen. For cell wall staining, hyphae were stained with 10 μg/mL CFW (Sigma-Aldrich, St. Louis, MO, USA) for 5 min as described [92]. Samples were examined for CMAC and CFW staining signals with an Olympus BX53 epifluorescence microscope (Olympus, Tokyo, Japan) or Zeiss LSM880 confocal microscope (Carl Zeiss, Jena, Germany).

## Figures and Tables

**Table 1:** Strains of *Fusarium graminearum* used in this study.

Strain	Brief Description	Reference
PH-1	Wild type	[40]
FY6	*Fgyck1* deletion mutant of PH-1	[7]
PHLA1	Transformant of PH-1 expressing LifeAct-GFP	[41]
FYC1	Complementary strain of *Fgyck1* mutant FY6	This study
RGY3, RGY5	GFP-*FgYCK1* transformant of PH-1	This study
S1-S25	Spontaneous suppressors of FY6	This study
FB5	*Fgbni4* deletion mutant of PH-1	This study
FB18	*Fgbni4* deletion mutant of PH-1	This study
FBC9	complementary strain of *Fgbni4* mutant FB5	This study
RC11	*FgBNI4*^R699C^ transformant of PH-1	This study
LF41	*FgBNI4*^ΔCT^ transformant of PH-1	This study
RCY14	*Fgyck1 FgBNI4*^R699C^ mutant	This study
LFY5	*Fgyck1 FgBNI4*^ΔCT^ mutant	This study
DK21, DK41	*Fgyck1 Fgbni4* double mutants	This study
FYLA1, FYLA3	LifeAct-GFP transformants of FY6	This study
PHSP26	*FgSPA2*-GFP transformant of PH-1	This study
FYSP21, FYSP22	*FgSPA2*-GFP transformants of FY6	This study
PHR12, PHR19	Transformants of PH-1 expressing GFP-*FgRHO1*	This study
FYR2	GFP-*FgRHO1* transformant of FY6	This study
DKR1, DKR2	GFP-*FgRHO1* transformants of DK21	This study

**Table 2:** Vegetative growth, conidiation, and virulence of *F. graminearum* strains.

Strain	Growth Rate (mm/Day) ^a^	Conidiation (×10^4^ Conidia/mL) ^b^	Disease Index ^c^
PH-1 (WT)	11.3 ± 0.5 ^A^*	123.8 ± 7.7 ^A^	8.8 ± 1.6 ^A^
FY6 (*Fgyck1*)	1.8 ± 0.0 ^C^	0	0
FYC1 (*Fgyck1*/*FgYCK1*)	11.2 ± 0.2 ^A^	129.1 ± 8.7 ^A^	8.8 ± 1.5 ^A^
RCY14 (*Fgyck1 FgBNI4*^R699C^)	3.0 ± 0.1 ^B^	45.2 ± 9.6 ^B^	0
LFY5 (*Fgyck1 FgBNI4*^ΔCT^)	3.0 ± 0.1 ^B^	46.4 ± 8.0 ^B^	0
DK21 (*Fgyck1 Fgbni4*-21)	3.2 ± 0.0 ^B^	45.8 ± 5.8 ^B^	0
DK41 (*Fgyck1 Fgbni4*-41)	3.1 ± 0.0 ^B^	46.6 ± 5.8 ^B^	0

^a^ Average radial growth per day on PDA plates. ^b^ Conidiation in 5-day-old CMC cultures. ^c^ Diseased spikelets per wheat head at 14 dpi. * Mean and standard deviation were calculated with data from three independent measurements. Different letters (A–C) indicate significant differences by Duncan’s multiple rage test (*p* = 0.05).

**Table 3:** Mutations identified in suppressor strains of *Fgyck1* mutant by whole genome sequencing.

Suppressor	Predicted Gene	Yeast Homolog	Nucleotide Change	Amino Acid Changes
S8	FGRRES_10104	*IES4*	C^568^AA to TAA	Q190 *
S12	FGRRES_07218	*BNI4*	G^889−1^T to AT (intron 1)	S297 fs ^IR^
	FGRRES_17518	*MSS4*	A^1058^ to C	N353T
S21	FGRRES_03646	*TNA1*	G^238^AT to AAT	D80N
	FGRRES_17597	*TOK1*	G^160^GT to CGT	G54R
S22	FGRRES_06258	none	ΔG^1473^A^1474^	N492 fs
S23	FGRRES_07218	*BNI4*	ΔC^2134^T^2135^	L712 fs
S24	FGRRES_03646	*TNA1*	G^238^AT to AAT	D80N
	FGRRES_09715	*MIG1*	ΔCTC^920–922^	DP307
S25	FGRRES_03646	*TNA1*	G^238^AT to AAT	D80N
	FGRRES_03242	none	ΔCAT^1113–1115^	DI371

* stop codon; fs, frame shift; ^IR^, intron retention; Δ, deletion.

## Data Availability

The data that support the findings of this study are available in the Supplementary Material of this article.

## Supplementary Materials

The following supporting information can be downloaded at: https://www.mdpi.com/article/10.3390/ijms23169106/s1.
