# Supplementary material for: Stage-Specific Genetic Interaction between FgYCK1 and FgBNI4 during Vegetative Growth and Conidiation in Fusarium graminearum
Source: Int J Mol Sci. 2022 Aug 14;23(16):9106. doi: 10.3390/ijms23169106 (PMC9408904; doi:10.3390/ijms23169106)
Supplement: Supplementary file 1 [file ijms-23-09106-s001.zip › Figure S1.pdf]

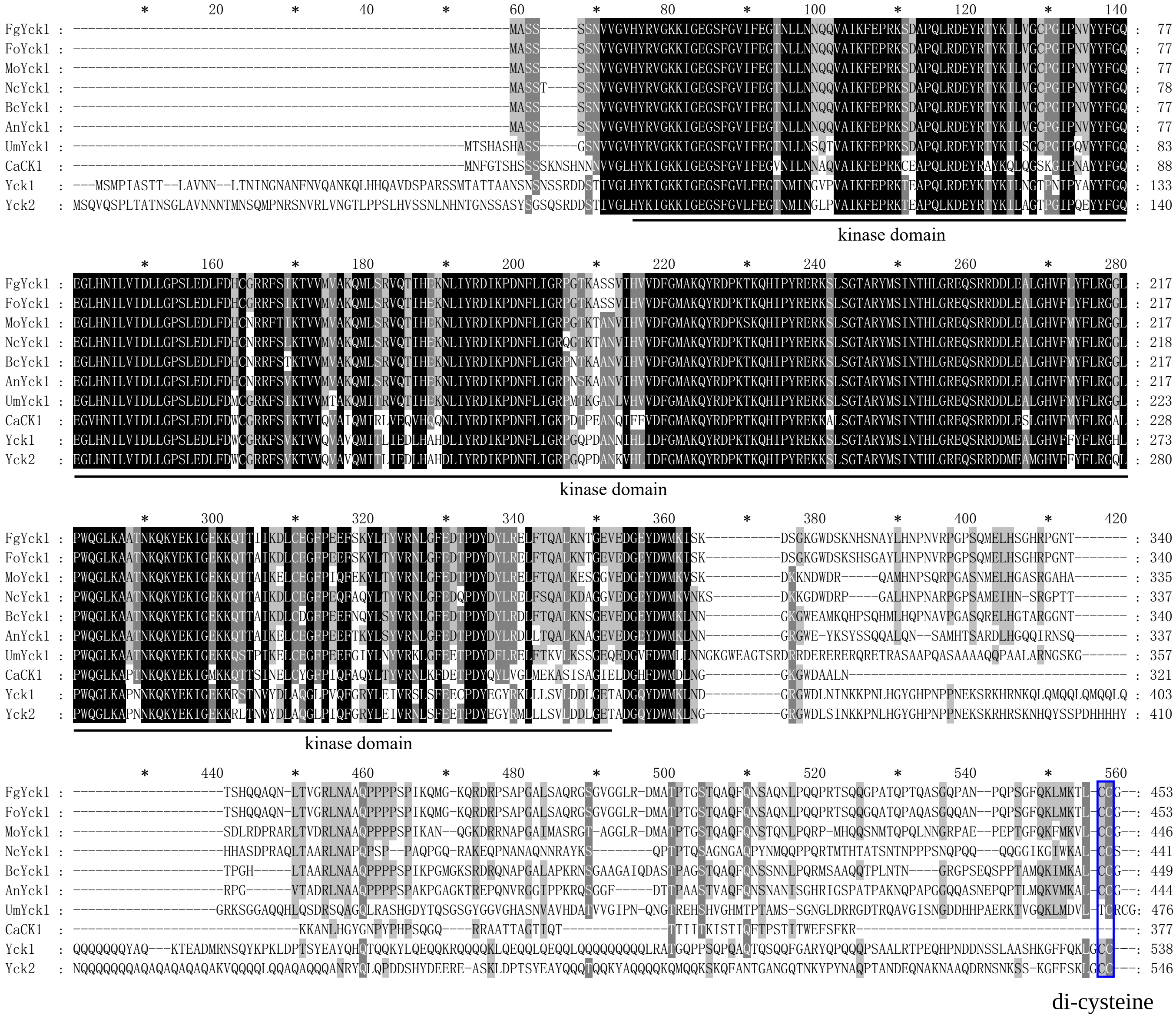
FgYck1
FoYck1
MoYck1
NcYck1
BcYck1
AnYck1
UmYck1
CaCK1
Yck1
Yck2
kinase domain
kinase domain
kinase domain
di-cysteine
