# Supplementary material for: Stage-Specific Genetic Interaction between FgYCK1 and FgBNI4 during Vegetative Growth and Conidiation in Fusarium graminearum
Source: Int J Mol Sci. 2022 Aug 14;23(16):9106. doi: 10.3390/ijms23169106 (PMC9408904; doi:10.3390/ijms23169106)
Supplement: Supplementary file 1 [file ijms-23-09106-s001.zip › Table S1.pdf]

Table S1 Phenotypes of the spontaneous suppressor strains.

Strain	Growth rate (mm/day)[a]	Conidiation ($\times 10^4$ conidia/mL)[b]	Conidial length (μm)[c]	Conidial septation (septa/conidium)[d]
PH-1	11.1±0.4	126.4±7.2	46.9±2.1	4.2±0.7
FY6 (*Fgyck1*)	1.6±0.2	0	0	0
S1	1.9±0.1	1.4±0.6	24.5±2.4	2.6±0.8
S2	4.3±0.2	1.3±0.5	28.4±4.2	2.8±0.6
S3	4.4±0.2	6.3±2.4	28.0±6.3	2.3±0.8
S4	3.2±0.1	0	0	0
S5	4.7±0.4	0	0	0
S6	3.7±0.3	3.6±1.5	29.5±6.5	2.8±1.2
S7	3.6±0.2	1.8±1.1	28.7±7.6	2.7±0.9
S8*	3.2±0.1	1.5±0.6	26.7±5.1	2.3±0.6
S9	2.0±0.1	0	0	0
S10	3.3±0.1	3.9±1.6	27.2±3.7	2.9±0.3
S11	2.9±0.1	8.6±3.5	23.9±2.1	2.3±1.0
S12*	4.0±0.1	22.9±5.6	28.2±4.9	2.5±0.9
S13	4.4±0.3	14.4±8.0	24.2±1.1	2.7±0.9
S14	3.4±0.2	4.6±2.2	27.2±3.4	2.6±0.5
S15	4.6±0.1	2.67±1.3	24.9±2.3	2.5±0.6
S16	2.5±0.1	0	0	0
S17	4.2±0.1	0	0	0
S18	3.1±0.2	0	0	0
S19	4.2±0.5	0	0	0
S20	2.8±0.1	0	0	0
S21*	3.7±0.2	2.1±0.9	24.7±2.5	2.3±0.8
S22*	2.4±0.1	0	0	0
S23*	4.5±0.1	8.5±2.9	29.4±4.2	2.7±1.0
S24*	4.6±0.1	9.9±3.0	20.9±1.4	2.0±0.7
S25*	3.9±0.1	0	0	0

* indicates suppressor strains selected for genome sequencing.

[a] average radial growth per day on PDA plates.

[b] conidiation in 5-day-old CMC cultures.

[c], [d] more than 30 conidia cells were examined.

Mean and standard deviation were calculated from three biological replicates.
