# Supplementary material for: Stage-Specific Genetic Interaction between FgYCK1 and FgBNI4 during Vegetative Growth and Conidiation in Fusarium graminearum
Source: Int J Mol Sci. 2022 Aug 14;23(16):9106. doi: 10.3390/ijms23169106 (PMC9408904; doi:10.3390/ijms23169106)
Supplement: Supplementary file 1 [file ijms-23-09106-s001.zip › Table S2.pdf]

Table S2. Mutations in *FgBNI4* and *FgTNA1* identified in spontaneous suppressors of *Fgyck1* mutant by direct sequencing of PCR products.

Suppressor strains	*FgBNI4*		*FgTNA1*	
	**Nucleotide change**	**Amino acid changes**	**Nucleotide change**	**Amino acid changes**
S1	nd	nd	nd	nd
S2	nd	nd	nd	nd
S3	C^{1468}GA to TGA	R490*	nd	nd
S4	nd	nd	nd	nd
S5	nd	nd	nd	nd
S6	nd	nd	nd	nd
S7	C^{2095}GC to TGC	R699C	nd	nd
S9	nd	nd	nd	nd
S10	$\Delta CT^{2051\text{-}2052}$	P684fs	nd	nd
S11	nd	nd	nd	nd
S13	nd	nd	nd	nd
S14	nd	nd	nd	nd
S15	$\Delta AC^{1617\text{-}1618}$	R539fs	nd	nd
S16	nd	nd	nd	nd
S17	nd	nd	nd	nd
S18	nd	nd	nd	nd
S19	nd	nd	nd	nd
S20	nd	nd	nd	nd

* stop codon; fs, frame shift; nd, not detected; Δ, deletion.
