# Supplementary material for: Stage-Specific Genetic Interaction between FgYCK1 and FgBNI4 during Vegetative Growth and Conidiation in Fusarium graminearum
Source: Int J Mol Sci. 2022 Aug 14;23(16):9106. doi: 10.3390/ijms23169106 (PMC9408904; doi:10.3390/ijms23169106)
Supplement: Supplementary file 1 [file ijms-23-09106-s001.zip › Table S3.pdf]

Table S3 Phenotypes of the *Fgbni4* mutant in growth, conidiation and plant infection.

Strain	Growth rate (mm/day)[a]	Conidiation ($\times 10^4$ conidia/mL)[b]	Disease index[c]
PH-1 (WT)	10.9±0.3[A*]	101.7±13.0[A]	8.9±1.3[A]
FB5 (*Fgbni4*)	10.4±0.4[A]	108.3±19.2[A]	7.7±1.5[A]
FBC9 (*Fgbni4*/*FgBNI4*)	11.0±0.2[A]	99.0±13.8[A]	9.4±1.7[A]

[a] Average radial growth per day on PDA plates.

[b] Conidiation in 5-day-old CMC cultures.

[c] Diseased spikelets per wheat head at 14 dpi.

* Mean and standard deviation were calculated from three independent replicates. Different letters indicate significant differences by Duncan's multiple rage test (p=0.05).
