# Supplementary material for: Stage-Specific Genetic Interaction between FgYCK1 and FgBNI4 during Vegetative Growth and Conidiation in Fusarium graminearum
Source: Int J Mol Sci. 2022 Aug 14;23(16):9106. doi: 10.3390/ijms23169106 (PMC9408904; doi:10.3390/ijms23169106)
Supplement: Supplementary file 1 [file ijms-23-09106-s001.zip › Table S4.pdf]

Table S4. Primers used in this study.

Name	Sequence (5'-3')	Application
B1F	TCGCTGTTTCTCCTGTGTCC	*FgBNI4* deletion
B2R	TTGACCTCCACTAGCTCCAGCCAAGCCAAGTTCCCGCATACTGGTGG	*FgBNI4* deletion
B3F	GAATAGAGTAGATGCCGACCGCGGGTTGCGACATTCTCTCCTCGCAT	*FgBNI4* deletion
B4R	GCAGCTTTCCCCAGATGAGT	*FgBNI4* deletion
B5F	AATCAACATGGCCGCTCTCA	*FgBNI4* deletion
B6R	CATCGTAGGCCTCGTCAGTC	*FgBNI4* deletion
B7F	TCCCTGGGCCGATAACCTTA	*FgBNI4* deletion
B8R	GACGTCATTGTCGACATGCG	*FgBNI4* deletion
HT/F	GATGTAGGAGGGCGTGGATATGTCCT	*FgBNI4* deletion
HY/R	GTATTGACCGATTCCTTGCGGTCCGAA	*FgBNI4* deletion
YG/F	GGCTTGGCTGGAGCTAGTGGAGGTCAA	*FgBNI4* deletion
HT/R	AACCCGCGGTCGGCATCTACTCTATTC	*FgBNI4* deletion
H852	AACTCACCGCGACGTCTGTC	*FgBNI4* deletion
H850	TTGTCCGTCAGGACATTGTT	*FgBNI4* deletion
H855R	GCTGATCTGACCAGTTGC	*FgBNI4* deletion
H866F	GTCGATGCGACGCAATCGT	*FgBNI4* deletion
FgBNI4/CF	AGGGAACAAAAGCTGGGTACCCCTTGTCCCCTGCATGCTTA	generation of complementation construct for *Fgbni4* mutant
FgBNI4/CR	GAACAGCTCCTCGCCCTTGCTCACGCAGCTTTCCCCAGATGAGT	generation of complementation construct for *Fgbni4* mutant
Y1F	GCTGTTCCCTAGCAGTCCAG	*FgYCK1* deletion
Y2R	CAGATACGGCAGAGAAATCGCAACCTCACGGTAGTGAACGCCTACG A	*FgYCK1* deletion
Y3F	GTTTAGATTCCAAGTGTCTACTGCTGGCGCCAGCTAACCCTCAGCCTA	*FgYCK1* deletion
Y4R	CGCATCTTCGATCATTCTTCATCAA	*FgYCK1* deletion
Y5F	CAATCTACTCAACAACCAGCAAGTT	*FgYCK1* deletion
Y6R	CCTTTCCCGAATCCTTAGAGATCTT	*FgYCK1* deletion
Y7F	CGTACAGTACGCAACTAGAAAAGTG	*FgYCK1* deletion
Y8R	TGAGACCAACCAGATTGAGAGC	*FgYCK1* deletion
GEN/F	GAGGTTGCGATTTCTCTGCCGTATCTG	*FgYCK1* deletion
GEN/R	GCCAGCAGTAGACACTTGGAATCTAAAC	*FgYCK1* deletion
G850	TCGGCTATGACTGGGCACAACA	*FgYCK1* deletion
G852	GAGCGGCGATACCGTAAAGCAC	*FgYCK1* deletion
G855/R	TGTTGGGTTTGAGCTAGGTGGG	*FgYCK1* deletion
G866/F	GAATGGTCAAATCAAACTGCTAGATAT	*FgYCK1* deletion
FgYCK1/CF	CGACTCACTATAGGGCGAATTGGGTACTCAAATTGGCGACAAGAACC TTGAATCTCAAC	generation of complementation construct for *Fgyck1* mutant

FgYCK1/CR	CTTTATAATCACCGTCATGGTCTTTGTAGTCTCGATCATTCTTCATCAAGCAGA	generation of complementation construct for *Fgyck1* mutant
P1F	GGCCGATCATTTGTTGGAATCTTC	generation of *FgBNI4*^{R699C} transformants
P1R	GCAGTCGTACTCCTGGCTAG	generation of *FgBNI4*^{R699C} transformants
P2F	CTAGCCAGGAGTACGACTGC	generation of *FgBNI4*^{R699C} transformants
P2R	CAGATACGGCAGAGAAATCGCAACCTCTCTGTAAATCACTTTCAACGGCAT	generation of *FgBNI4*^{R699C} transformants
P3F	GTTTAGATTCCAAGTGTCTACTGCTGGCTCTGGTGTCTGATCCATACTTGATA	generation of *FgBNI4*^{R699C} transformants
P3R	CTCCTTCATCCATTCTGTATCGTCT	generation of *FgBNI4*^{R699C} transformants
S1F	AGGGCGCAAGTCTAAGAGC	generation of *FgBNI4*$^{\Delta CT}$ transformants
S1R	TGCGCAAGCAGAGGAGTCAATCGATTGCACGT	generation of *FgBNI4*$^{\Delta CT}$ transformants
S2F	CGATTGACTCCTCTGCTTGCGCAACAGATCAAGG	generation of *FgBNI4*$^{\Delta CT}$ transformants
S2R	TTGACCTCCACTAGCTCCAGCCAAGCCCGTTCCCAGTTTCAACGGTGA	generation of *FgBNI4*$^{\Delta CT}$ transformants
S3F	CGTCCGCAATGTGTTATTAAGTCGACCTCAATGGAGCGGACATAACG	generation of *FgBNI4*$^{\Delta CT}$ transformants
S3R	TTCTGGTAAGCATGCGCCAGG	generation of *FgBNI4*$^{\Delta CT}$ transformants
GFP/1F	GTAGGAACCCAATCTTCAAACTCGAGATGGTGAGCAAGGGCGAGGA	generation of GFP-*FgRHO1* construct with RP27 promoter
GFP/2R	TCCTCCTCCTCCTCCTCCTCCTCCTCCCTTGTACAGCTCGTCCATGCCGA	generation of GFP-*FgRHO1* construct with RP27 promoter
GFP-RHO1/F	GGAGGAGGAGGAGGAGGAGGAGGAGGAGGATCC ATGGCTGAGATCCGCCGAA	generation of GFP-*FgRHO1* construct with RP27 promoter
GFP-RHO1/R	TGATTTCAGTAACGTTAAGTGGATCCAAACGAAAAGATTTATGGCCTGACC	generation of GFP-*FgRHO1* construct with RP27 promoter
FgSPA2/F	AGGGAACAAAAGCTGGGTACCCCCATAAACACTTGAGGCCTCT	generation of GFP-*FgSPA2* construct
FgSPA2/R	GAACAGCTCCTCGCCCTTGCTCACCGAAAAGTCATCAGGTCGTCCG	generation of GFP-*FgSPA2* construct
FgBNI4/TF	AACAATTAACAAAAGCGACTCTCGT	DNA sequencing of *FgBNI4* gene
FgBNI4/TR	GAGACTGGTTAGAGTAATTGAACGC	DNA sequencing of *FgBNI4* gene
FgTNA1/TF	ATAATACCCAGCGATGTCTTGTGTA	DNA sequencing of *FgTNA1* gene
FgTNA1/TR	CTCGTGAGGTGTGGAGTTATGATAT	DNA sequencing of *FgTNA1* gene
