# Supplementary figures and images for: Stage-Specific Genetic Interaction between FgYCK1 and FgBNI4 during Vegetative Growth and Conidiation in Fusarium graminearum
Source: Int J Mol Sci. 2022 Aug 14;23(16):9106. doi: 10.3390/ijms23169106 (PMC9408904; doi:10.3390/ijms23169106)

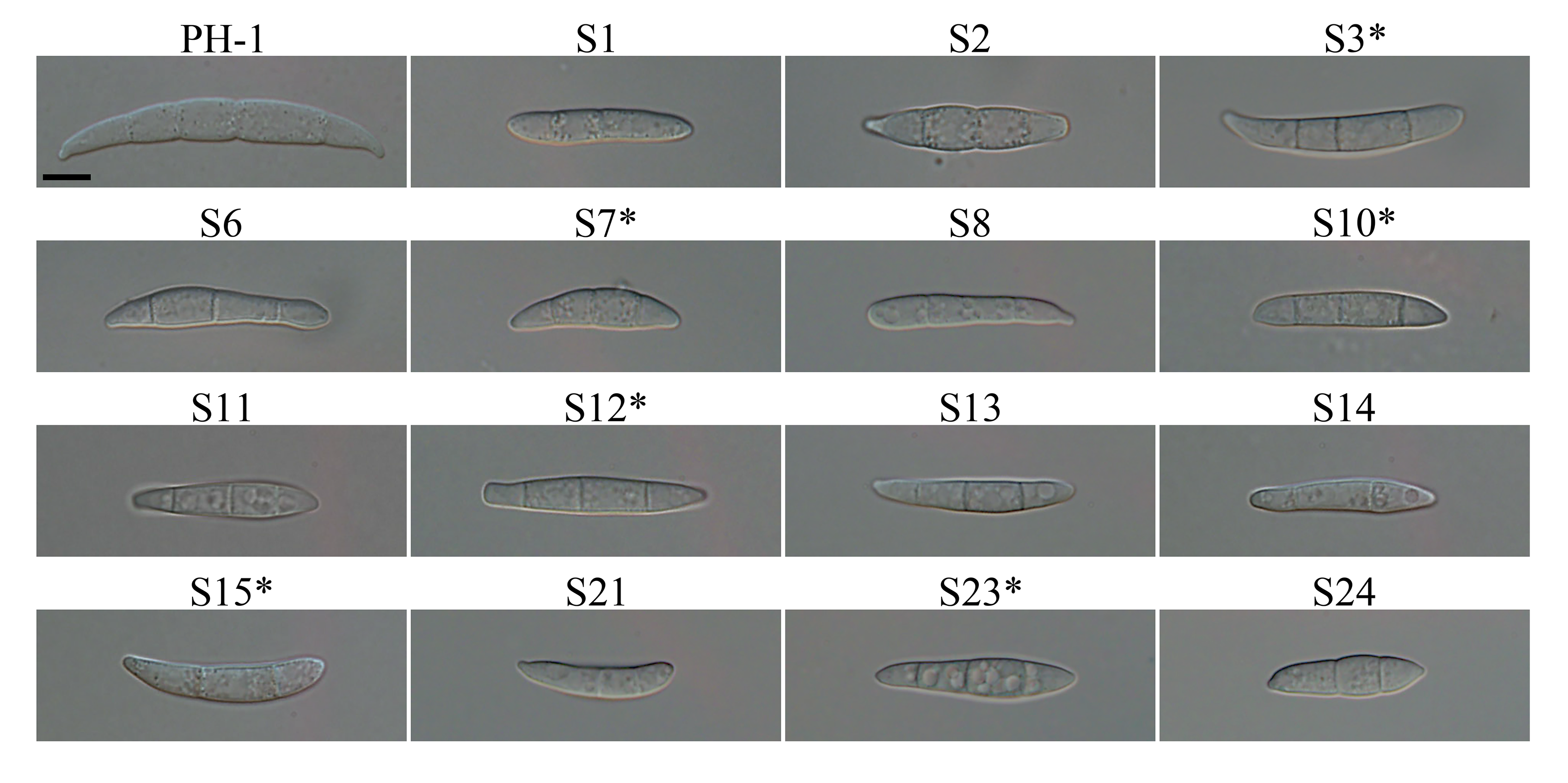

Supplement: Supplementary file 1 [file ijms-23-09106-s001.zip › Figure S2.tif]

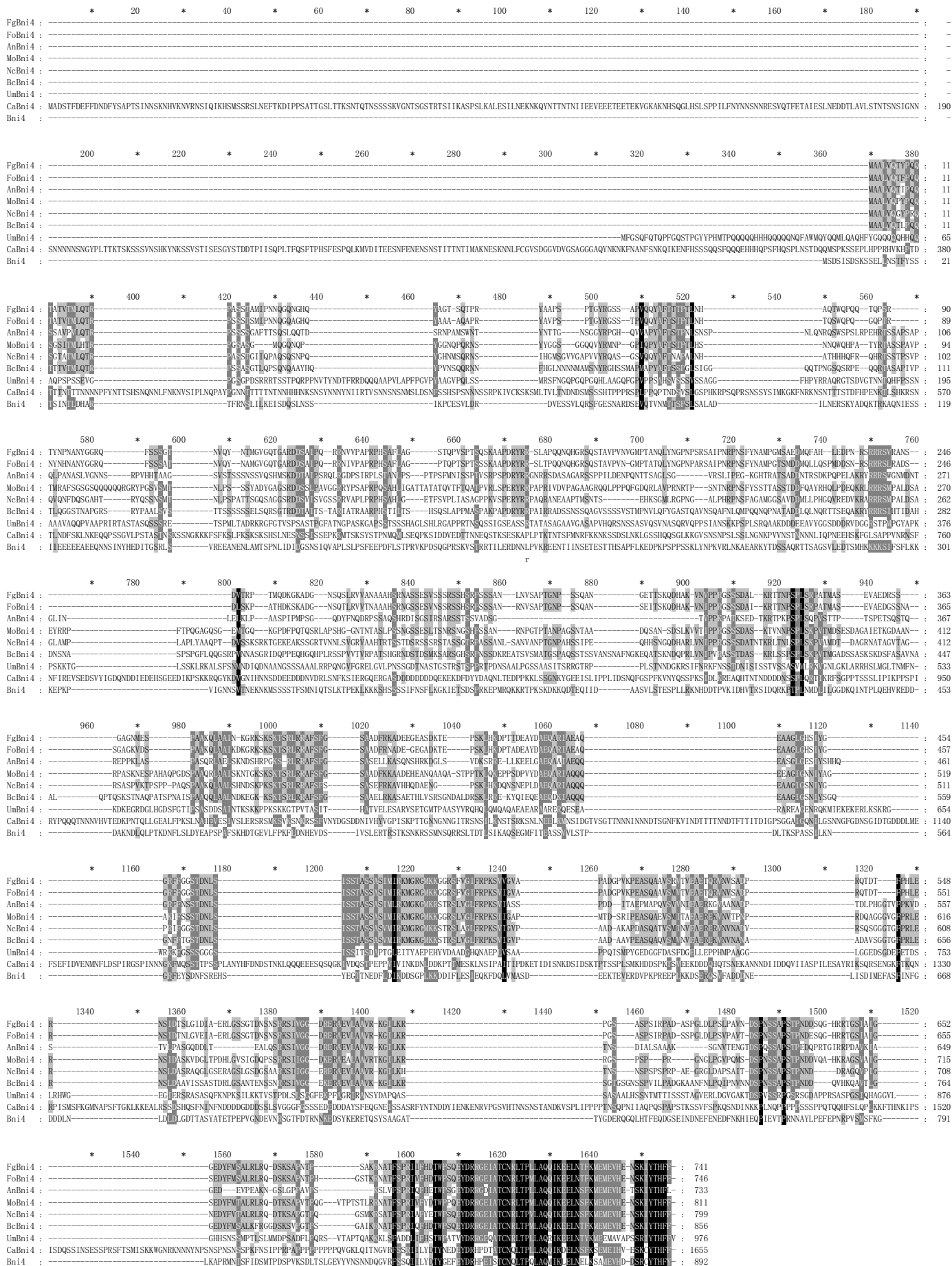

Supplement: Supplementary file 1 [file ijms-23-09106-s001.zip › Figure S3.pdf]

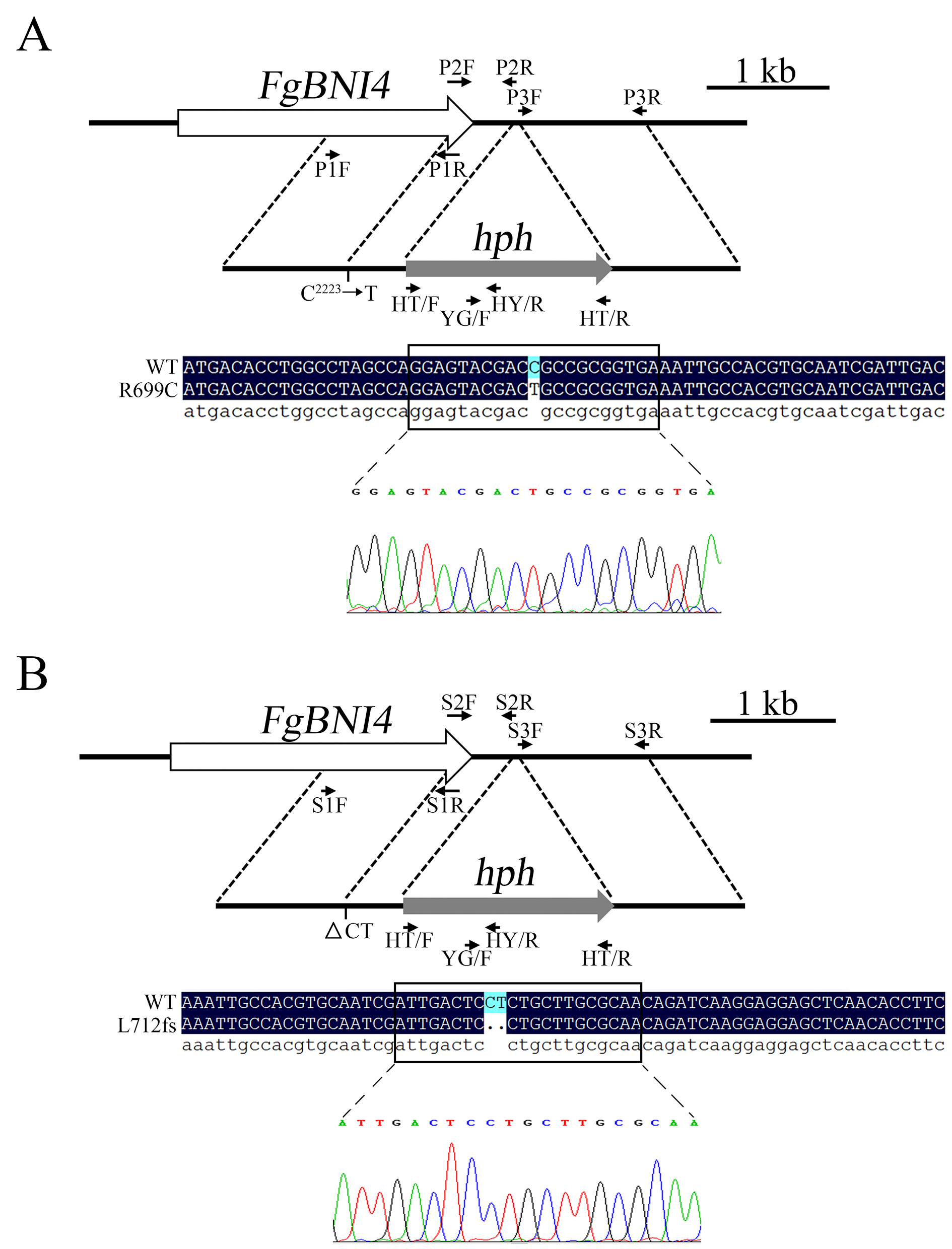

Supplement: Supplementary file 1 [file ijms-23-09106-s001.zip › Figure S4.tif]

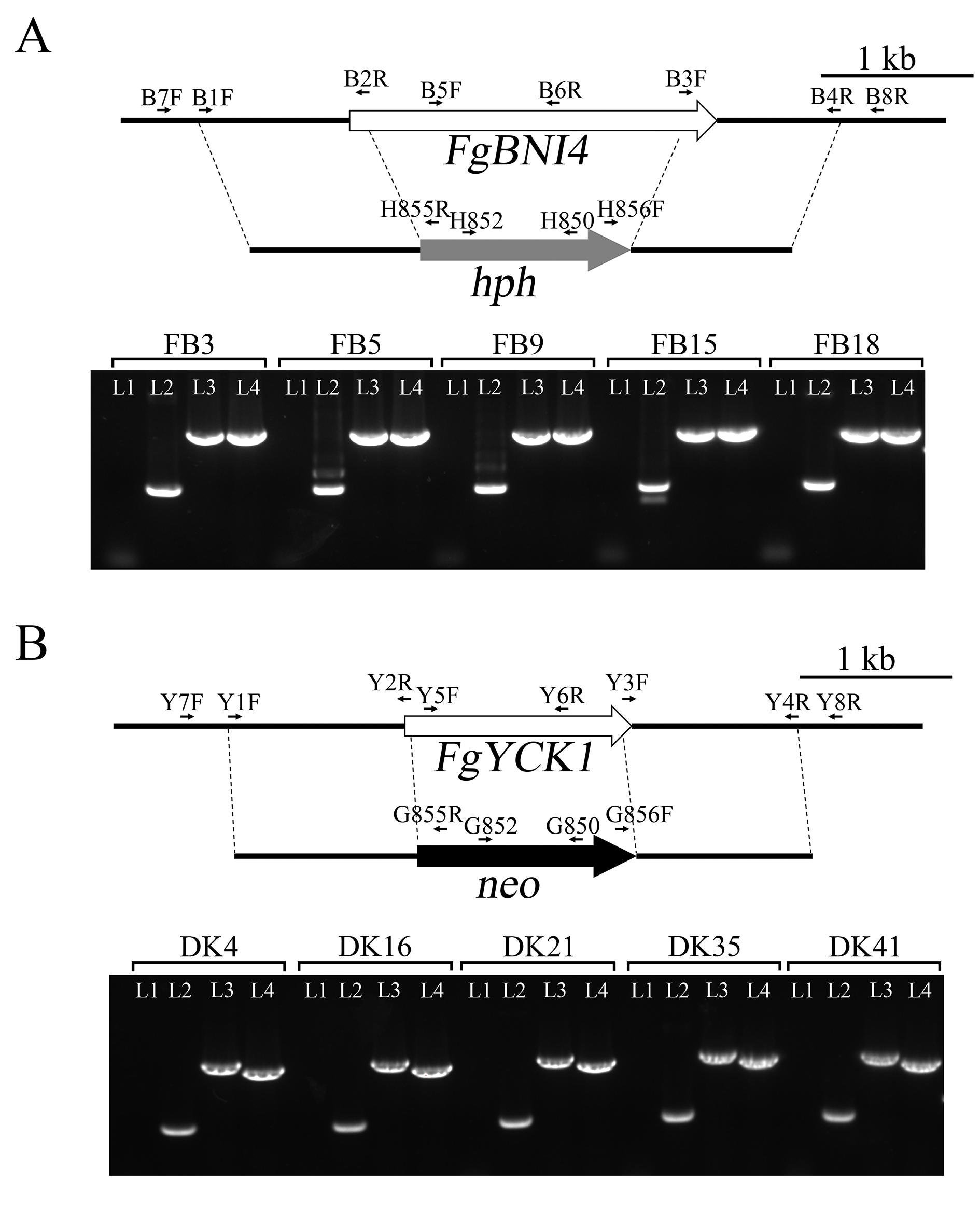

Supplement: Supplementary file 1 [file ijms-23-09106-s001.zip › Figure S5.tif]

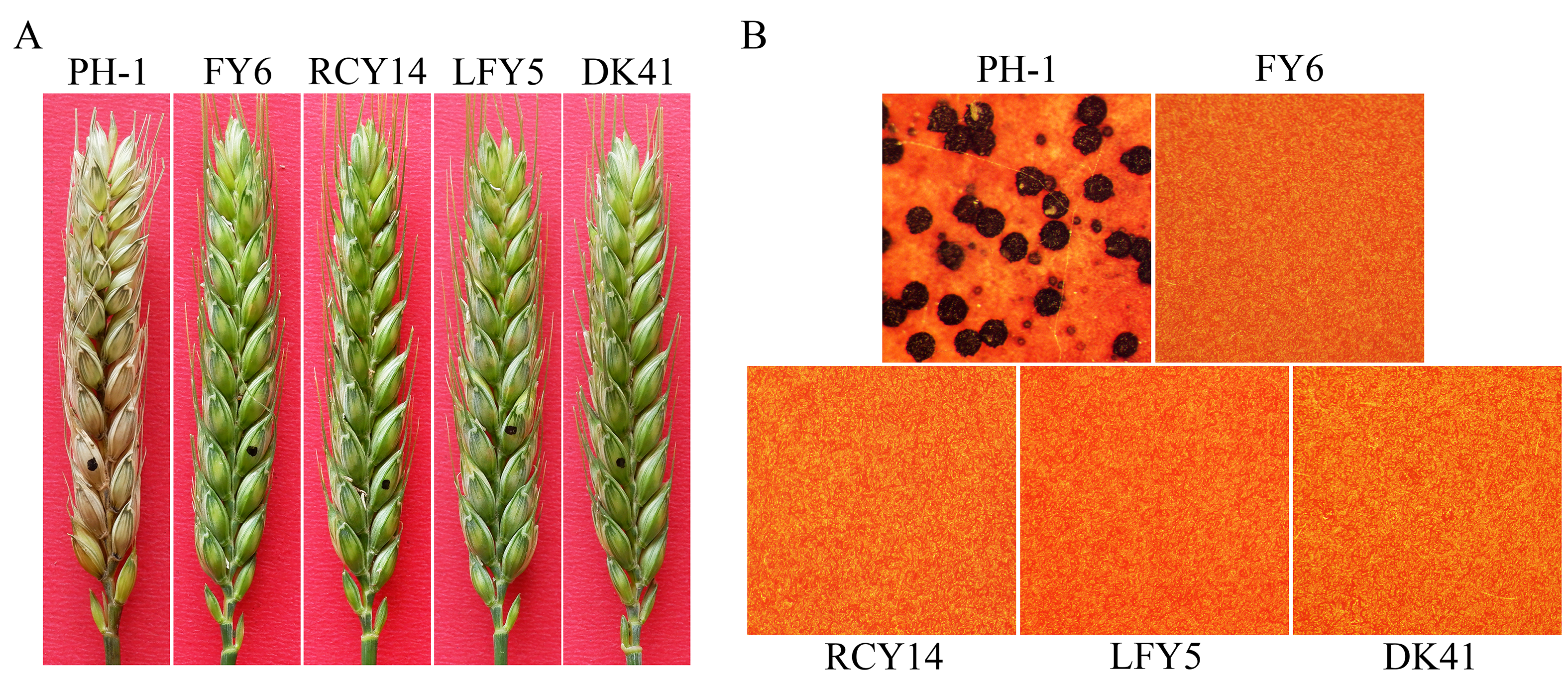

Supplement: Supplementary file 1 [file ijms-23-09106-s001.zip › Figure S6.tif]

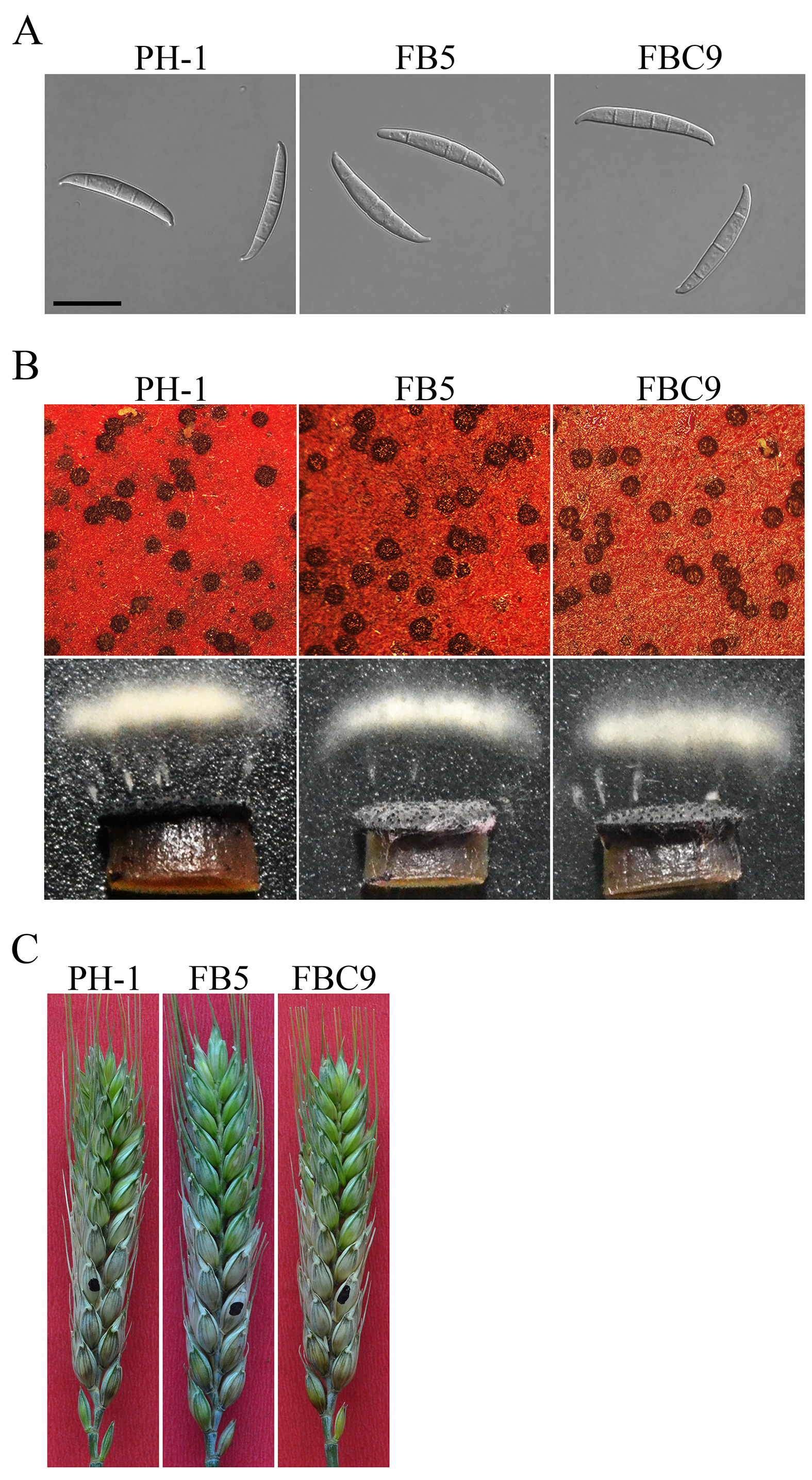

Supplement: Supplementary file 1 [file ijms-23-09106-s001.zip › Figure S7.tif]
